# Biomimetic design of bioartificial scaffolds for the *in vitro* modelling of human cardiac fibrosis

**DOI:** 10.3389/fbioe.2022.983872

**Published:** 2022-11-24

**Authors:** Mattia Spedicati, Gerardina Ruocco, Alice Zoso, Leonardo Mortati, Andrea Lapini, Andrea Delledonne, Carla Divieto, Veronica Romano, Clotilde Castaldo, Franca Di Meglio, Daria Nurzynska, Irene Carmagnola, Valeria Chiono

**Affiliations:** ^1^ Department of Mechanical and Aerospace Engineering, Politecnico di Torino, Torino, Italy; ^2^ POLITO Biomedlab, Politecnico di Torino, Torino, Italy; ^3^ Interuniversity Center for the Promotion of the 3Rs Principles in Teaching and Research, Pisa, Italy; ^4^ Istituto Nazionale di Ricerca Metrologica (INRIM), Torino, Italy; ^5^ Department of Chemistry, Life Science and Environmental Sustainability, University of Parma, Parma, Italy; ^6^ Department of Public Health, University of Naples “Federico II”, Napoli, Italy; ^7^ Department of Medicine, Surgery and Dentistry “Scuola Medica Salernitana”, University of Salerno, Salerno, Italy

**Keywords:** bioartificial scaffold, cardiac fibrosis, *in vitro* model, poly(caprolactone), gelatin, extracellular matrix

## Abstract

*In vitro* models of pathological cardiac tissue have attracted interest as predictive platforms for preclinical validation of therapies. However, models reproducing specific pathological features, such as cardiac fibrosis size (i.e., thickness and width) and stage of development are missing. This research was aimed at engineering 2D and 3D models of early-stage post-infarct fibrotic tissue (i.e., characterized by non-aligned tissue organization) on bioartificial scaffolds with biomimetic composition, design, and surface stiffness. 2D scaffolds with random nanofibrous structure and 3D scaffolds with 150 µm square-meshed architecture were fabricated from polycaprolactone, surface-grafted with gelatin by mussel-inspired approach and coated with cardiac extracellular matrix (ECM) by 3 weeks culture of human cardiac fibroblasts. Scaffold physicochemical properties were thoroughly investigated. AFM analysis of scaffolds in wet state, before cell culture, confirmed their close surface stiffness to human cardiac fibrotic tissue. Following 3 weeks culture, biomimetic biophysical and biochemical scaffold properties triggered the activation of myofibroblast phenotype. Upon decellularization, immunostaining, SEM and two-photon excitation fluorescence microscopy showed homogeneous decoration of both 2D and 3D scaffolds with cardiac ECM. The versatility of the approach was demonstrated by culturing ventricular or atrial cardiac fibroblasts on scaffolds, thus suggesting the possibility to use the same scaffold platforms to model both ventricular and atrial cardiac fibrosis. In the future, herein developed *in vitro* models of cardiac fibrotic tissue, reproducing specific pathological features, will be exploited for a fine preclinical tuning of therapies.

## 1 Highlights

1) Myocardial infarction, a global issue, causes cardiac tissue adverse remodeling.
2) Fibroblasts activation, tissue stiffening, alignment loss typify cardiac fibrosis.
3) *In vitro* models are useful for preclinical validation of new regenerative therapies.
4) Cellularized cardiac biomatrix-coated 2D and 3D scaffolds mimic human fibrotic tissue at different thicknesses.

## 2 Introduction

Myocardial infarction (MI) is the main cause of mortality and morbidity worldwide and responsible for more than 50% of cardiovascular deaths (World Health Organization, 2017). The irreversible loss of billions of cardiomyocytes (CMs) (Mozaffarian, 2015; Sutton and Sharpe, 2000) starts a wound healing process through an early inflammatory stage, subsequent recruitment and proliferation of cardiac fibroblasts (CFs) and their activation into myofibroblasts (MyoFs), mediated by profibrotic signals. CFs differentiation into MyoFs is marked by the expression of α-smooth muscle actin (α-SMA) (Yang et al., 2002; Czubryt, 2012; van Nieuwenhoven and Turner, 2013; Deng et al., 2017) and an over-deposition of extracellular matrix (ECM) proteins, especially type I and type III collagens, fibronectin, laminin α_1_ and tenascin-X (Dale Brown et al., 2005; Baudino et al., 2006; Leask, 2010; Castaldo et al., 2013). The ECM components increase in concentration and appear disorganized in their arrangement, causing CM hypertrophy and alignment loss during the early fibrotic stage (Factor et al., 1991; Shirani et al., 2000). Due to these structural changes, scar tissue is stiffer than healthy tissue, ranging from dozens of kPa to few MPa (Chelnokova et al., 2016; Sadeghi et al., 2017). Currently, there is no effective clinical treatment able to contrast fibrosis and to recover myocardial functionality, therefore heart transplantation remains the only clinical treatment for end-stage heart failure (Chaudhuri et al., 2017). However, several cardiac regenerative medicine strategies are under investigation (Giacca, 2020), such as cell transplantation (Menasché, 2018), and cell reprogramming approaches aimed at inducing CM proliferation (Chen et al., 2008; Wang et al., 2017) or the trans-differentiation of fibroblasts into CMs (Paoletti, Divieto and Chiono, 2018; Paoletti et al., 2020). Preclinical validation of new therapies through 2D cell culture do not reproduce the 3D cell-cell and cell-ECM interactions and oversimplify cardiac extracellular microenvironment. Instead, although *in vivo* animal models mimic the complexity of living organisms, they fail to replicate specific human physiology due to inter-species differences, resulting in limited predictivity (Mathur et al., 2016; Conant et al., 2017; Harrison, 2016). The use of human *in vitro* 3D pathological models could improve the reliability of drug preclinical validation and favor a reduction in animal experimentation, in agreement with the 3Rs principle (Reduction, Replacement, Refinement) (Sadeghi et al., 2017). Up to now, *in vitro* models of pathological cardiac tissue have been mainly engineered using hydrogel concentration and crosslinking degree (van der Valk et al., 2018) (Sang Bok Kim et al., 2011). For example, Zhao et al. (2014) designed an *in vitro* fibrotic cardiac tissue model from polyethylene glycol diacrylate (PEGDA) hydrogel functionalized with collagen and cellularized with adult rat CFs. PEGDA/collagen hydrogel stiffness influenced adult rat CFs activation, with CFs differentiating into MyoFs on stiff (∼40 kPa) substrates. Sadeghi et al. (2017) prepared a gelatin methacryloyl (GelMA)-based 3D hydrogel platform with physiological stiffness encapsulating co-cultures of neonatal rat CMs and CFs to evaluate the effect of TGF-β1 on CFs differentiation. The main limitations of the previous studies are the use of mouse or rat cardiac cells, which are not predictive of human cell response, and weak ability of hydrogels in providing structural cues. To address some of these limitations, “bioartificial” scaffolds, based on synthetic and natural polymers could provide biomimetic substrates with tailor-designed architecture, able to combine the cell recognition properties of proteins with the processability, shape stability, slow degradation rate and superior mechanical resistance of hydrogels (Ciardelli et al., 2005; Chiono et al., 2009; Chiono et al., 2014). For example, Kai et al. (2011) obtained rabbit CMs alignment on oriented electrospun PCL/gelatin nanofibrous scaffolds, mimicking ECM orientation of healthy myocardium. Castilho et al. (2017) reproduced cardiac tissue alignment by guiding cell arrangement on a poly (hydroxymethylglycolide-co-ε-caprolactone) (pHMGCL)-based scaffolds fabricated by melt electrospinning melt electrowriting combined with collagen hydrogel, obtaining an *in vitro* 3D model of healthy human cardiac tissue. Boffito et al. (2018) showed that square-meshed scaffolds based on an elastomeric synthesized polyurethane (PU), surface functionalized with laminin, were able to support human cardiac progenitor cells differentiation compared to the unfunctionalized PU scaffolds. Despite their potentialities, “bioartificial” scaffolds have never been exploited to engineer cardiac fibrosis *in vitro.*

In this work, we proposed a new platform of bioartificial scaffolds for the design of *in vitro* models of early-stage human cardiac fibrotic tissue with two different thicknesses, fabricating two substrates with completely different dimensional scale (2D and 3D scaffolds), mimicking the random organization, ECM composition (fibronectin, laminin, collagen I-III, tenascin) (Castaldo et al., 2013), and cell population (MyoFs differentiated from human cardiac fibroblasts) of the target pathological tissue. Novelty of this work mainly arises from the design of *in vitro* models mimicking specific cardiac fibrotic tissues (e.g., thickness and stage), aimed at preclinical validation of regenerative therapies for the specific clinical case. More in detail, main attention was addressed to the modelling of post-infarct left ventricle fibrotic tissue, consisting of pathological cardiac ECM with embedded ventricular human cardiac fibroblasts (v-HCFs) differentiated into MyoFs. Polycaprolactone (PCL) 2D and 3D scaffolds with non-aligned structure were prepared through solution electrospinning and melt-extrusion additive manufacturing (MEAM), respectively. Then, scaffolds were surface grafted with gelatin (G) to support v-HCFs activation and pathological cardiac ECM deposition. Each step of construct design was thoroughly characterized, by a variety of physicochemical and biological analyses.

Proof-of-concept studies of *in vitro* culture of cardiac fibroblasts isolated from human atrial samples (a-HCFs) on the same platforms were also performed to analyze the versatility of the approach in modelling other fibrosis types. Atrial fibrosis is generally a consequence of valvular defects, hypertension and aging, and has a key role in the development and persistence of atrial fibrillation (Pellman et al., 2010).

The reported results demonstrate that the proposed 2D and 3D models of early-stage cardiac fibrosis represent biomimetic platforms for future *in vitro* preclinical testing of new advanced therapies for cardiac regeneration.

## 3 Materials and methods

### 3.1 Materials

Polycaprolactone (M_w_ = 43,000 Da, PCL) was supplied by Polysciences; chloroform 99.8% and formic acid 98% were purchased from Sigma-Aldrich (Milan) and were used without further purification. 3,4-Dihydroxy-DL-phenylalanine (DOPA) and porcine gelatin (G) type A were obtained from Sigma-Aldrich (Milan).

### 3.2 Fabrication of 2D scaffolds

A 20% wt/v solution of PCL in a chloroform/formic acid mixture (70/30 v/v) was prepared. Firstly, PCL pellets were dissolved in chloroform under magnetic stirring at 200 rpm for 3 h. Then formic acid was added and the solution was magnetically stirred at 200 rpm for additional 40 min to obtain homogenous solution. Electrospun random mats were obtained employing electrospinning equipment (Linari Engineering S.r.l) with 5 mL glass syringe (21G needle), setting a voltage of 15 kV, a flow rate of 0.5 mL/h and a needle/collector distance of 15 cm. Nanofibers were collected on a flat collector covered with aluminum foil. For next physicochemical analysis and *in vitro* cell cultures, electrospun PCL membranes on 12 mm diameter culture slides were used.

### 3.3 Design and fabrication of 3D scaffolds

PCL porous scaffolds with square-meshed grid layers were produced by MEAM using *INVIVO* 3D printer (Rokit Healthcare, Republic of Korea) equipped with a 200 µm nozzle. Syringe temperature was set at 100°C to allow polymer melting. Air pressure, necessary for extrusion, was set at 650 kPa. Printing parameters as Print Speed (PS), Rotate Angle (RA) and Infill Density (FD) were set directly inside Creator K slicing software. A rectangular prism CAD model (20 mm × 20 mm × 0.7 mm) was designed with SolidWorks software® and then converted and exported in STL format. Scaffolds with 7 layers were fabricated using RA of 90° and FD of 70%, resulting in 150 µm average square grid size (Table S1). For physicochemical analyses and for *in vitro* cell cultures, scaffold samples with 7 mm × 7 mm × 0.7 mm were used.

**TABLE 1:** Contact angle values of PCL-based films, 2D and 3D scaffolds after each functionalization step.

Sample type	Contact angle value (°)		
	PCL	PCL/polyDOPA	PCL/polyDOPA/G
Film	76 ± 1	58 ± 5	58 ± 3
2D scaffold	141 ± 5	48 ± 8	40 ± 6
3D scaffold	133 ± 3	n.d	n.d

### 3.4 Films by solvent casting

PCL films were prepared *via* solvent casting technique, to be exploited in the physicochemical characterizations. In detail, 50 mL of 10% w/v PCL solution in chloroform was poured onto 11 cm diameter glass dish and then, placed under a vented hood to allow solvent evaporation.

### 3.5 Gelatin grafting

Gelatin was grafted on PCL scaffolds and films through a mussel inspired adhesive pre-coating (Carmagnola et al., 2020). This approach is versatile and can be applied to both inorganic and organic substrates. Besides, it requires mild processing conditions which minimize the risks for biomaterial alteration during the surface modification. Moreover, the approach can be applied on scaffolds with different geometries. One further advantage is the possibility to chemically graft molecules, such as bioactive proteins, on polyDOPA adhesive pre-coating using mild conditions, by incubating the polyDOPA-coated substrate in water-based protein solution, at room temperature and slightly alkaline pH, under moderate mechanical stirring. In this work, 2D and 3D PCL scaffolds and PCL films were incubated in DOPA solution (2 mg/mL in 10 mM Tris/HCl at pH 8.5) for 7 h at room temperature. The solution was kept under stirring at 100 rpm to promote DOPA oxidation and self-polymerization. Scaffolds were then washed with Tris/HCl buffer solution three times (PCL/polyDOPA). Subsequently, PCL samples were incubated in G solution (2 mg/mL in Tris/HCl at pH 8.5) for 16 h at room temperature (PCL/polyDOPA/G). Samples were then thoroughly washed with Tris/HCl buffer solution (pH 8.5) thrice and then, with distilled water for three times, to remove residual physically adsorbed G and Tris/HCl salts. Control 2D and 3D scaffolds were also prepared by G physical adsorption on PCL scaffolds (PCL/G), obtained by incubation of PCL scaffolds in G solutions.

### 3.6 Morphological characterization of scaffolds

The morphology of the exposed surface of electrospun mats and scaffolds prepared by MEAM was analyzed using a Scanning Electron Microscope (SEM, LEO 435VP). In the case of 3D scaffolds, samples were fractured in liquid nitrogen and sections were also analyzed. Samples were coated with a thin gold layer by using Agar Auto Sputter Coater instrument. SEM images were taken at different magnifications: ×1000, ×2000, and ×5000.

Electrospun membrane fiber diameter and pore size were evaluated by analyzing SEM images (×10000) by ImageJ software. To determine the average fiber size, 50 fibers were analyzed for each image and measurements were conducted in triplicate. Pore and filament size of 3D scaffolds prepared by MEAM were measured by analyzing optical microscopy images at different magnifications (×15, ×20) by ImageJ software.

### 3.7 3D Scaffolds porosity evaluations

Porosity percentage was measured by a gravimetric method through Eq. 1:

$$Porosity\;\left(\%\right)=\left(1-\frac{\rho_{\mathrm{scaffold}}}{\rho_{\mathrm{PCL}}}\right)∙100$$ (1)

where ρ_scaffold_ is scaffold density calculated by dividing scaffold weight by its volume, while ρ_PCL_ is PCL density as reported by Polysciences supplier $\left(1.145\;g/cm^{3}\right)$ . In the case of scaffolds prepared by MEAM, the porosity value was compared with theoretical porosity percentage, evaluated as a function of FD additive manufacturing parameter, according to Eq. 2:

Theoretical Porosity (%)=(100−FD) (2)

These analyses were performed in triplicate for each scaffold type.

### 3.8 Scaffold surface area estimation

The surface area estimation of 2D scaffolds was estimated by analyzing SEM images (×10000) through ImageJ software. Each 2D scaffold was approximated to a perfectly flat sample. In detail, by adjusting the threshold parameter, the area occupied by pores was calculated and subtracted to the total image area, obtaining the scaffold area per analyzed image. The process was repeated in triplicate on different images at the same magnification (×10000) and an average scaffold area value was calculated. Considering the size of 2D scaffolds (12 mm diameter) and of each analyzed image, the scaffold area for each sample was estimated proportionally. As nanofibrous 2D scaffolds were fixed on glass slides during *in vitro* cultures, only their exposed area was evaluated.

Surface area of 3D scaffolds (7 mm × 7 mm × 0.7 mm) was estimated from scaffold CAD models using measurement tool in Autodesk “Inventor”. Total area was calculated as single filament exposed surface area *per* number of filaments, according to fill density. In details, each filament was approximated to a cylinder and crossing area with perpendicular filaments in the adjacent layers was removed.

### 3.9 Quartz crystal microbalance

QCM-D QSense (Biolin Scientific, Finland) was used to evaluate the effectiveness of scaffold functionalization process with polyDOPA/G by reproducing every functionalization step. QCM-D was equipped with static module and gold sensor and temperature was set at 22°C. DOPA solution (300 µL) was placed on the micro balance using a micropipette and left for 7 h. Subsequently, to simulate the washing steps, DOPA solution was removed, and buffer solution was added and exchanged for three times after 5 min incubation. Finally, G solution (300 µL) was introduced and left for 16 h, and then final washes were performed, using firstly Tris/HCl solution for three times and then milliQ water for additional three times. During the analysis, 13 overtones were observed and data about frequency resonator (∆f) and dissipation energy (∆D) variation were acquired. Mass and thickness of deposited layers were calculated by QSense “Dfind” software. Considering different viscoelastic properties of both polyDOPA and G layers, two analysis models were applied: Sauerbrey model (suitable for rigid substrates) was chosen to calculate mass and thickness for polyDOPA coating, while Dfind “Smartfit” model (suggested for rigid thin layer and soft thick layer) was applied in order to correctly evaluate G layer properties.

### 3.10 Bicinchoninic acid assay

BCA Protein Assay Kit (Thermo Scientific Pierce) is a colorimetric method used for the detection and quantification of G grafted on 2D and 3D scaffolds (PCL/polyDOPA/G). The analysis was also conducted to demonstrate the efficacy of polyDOPA adhesive precoating in enhancing G grafting on scaffolds surface: PCL/G and PCL/polyDOPA/G samples were placed in the 24-multiwell plate and treated with 50 µL of diluent (phosphate buffered saline, PBS) and 400 µL of BCA Working Reagent. Scaffolds were then incubated for 30 min at 37°C. Finally, the absorbance was measured at 562 nm on a plate reader (Synergy HTX Multi-Mode Microplate Reader, BioTek). Based on a calibration curve, grafted G was quantified and expressed both as protein weight per scaffold and protein weight per exposed surface unit (µg/cm^2^). For each type of material, three samples were analyzed and data were reported as the average value ± standard deviation.

### 3.11 Contact angle measurements

Static contact angle of PCL, PCL/polyDOPA and PCL/polyDOPA/G films and scaffolds was measured at room temperature to evaluate the effect of surface modification on sample surface wettability (Wang et al., 2011). Moreover, to evaluate coating stability, static contact angle measurements were also performed on PCL, PCL/polyDOPA, PCL/G, PCL/polyDOPA/G films after their incubation at 37°C in PBS solution for 1, 3 and 7 days. The analysis was carried out using Drop Shape Analyzer equipped with Advanced software (KRÜSS GmbH—KRÜSS Scientific Instruments) selecting the sessile drop method. A drop of MilliQ water (2 µL) was placed on sample surface and the contact angle was sized. For each type of sample, measurements were performed at least five times at different locations right after drop deposition and 5 s later. Measurements were performed in triplicate. Static contact angles were reported as average values ± standard deviation.

### 3.12 Ventricular human cardiac fibroblasts culture and viability, cytotoxicity, and apoptosis assays

v-HCFs isolated from human ventricle and Fibroblasts Growth Medium-3 (FGM-3) were purchased from PromoCell. v-HCFs were maintained in FGM-3 composed of basal medium supplemented with 10% fetal calf serum, 1 ng/mL human basic Fibroblast Growth Factor and 5 μg/mL recombinant human insulin. Cells were maintained at 37°C in humidified atmosphere, 5% CO_2_.

Before cell seeding, 2D and 3D PCL, and PCL/polyDOPA/G scaffolds were disinfected by immersion in 70% v/v ethanol (EtOH) for 15 min, followed by rinsing in sterile phosphate buffered saline (PBS). Samples were then exposed to 15 min UV irradiation for each side and finally incubated overnight in 2X antibiotic-antimycotic solution (Life Technologies) in PBS, followed by PBS rinsing. Each scaffold was seeded with 25000 cells in a volume of 30 µL medium. After 2 h incubation, 500 µL FGM-3 medium was added to each well. Culture medium was replaced every 2–3 days with an equal volume of fresh medium. Cell viability was then analyzed after 1 and 7 days of culture by CellTiter-Blue^®^ Cell Viability Assay (Promega) and any potential biomaterials cytotoxicity was tested by CytoTox-ONE™ Homogeneous Membrane Integrity Assay (Promega). These analyses were conducted in biological triplicate.

### 3.13 Long-term *in vitro* cultures and decellularization protocol

v-HCFs and a-HCFs were seeded on 2D and 3D PCL/polyDOPA/G scaffolds as previously described and cultured up to 3 weeks, refreshing culture media every 48 h, to allow extracellular matrix secretion. Then, scaffolds were decellularized as described by Castaldo et al. (2013) by incubating samples for 1 min in 0.25% Triton (Sigma-Aldrich), 10 mM NH_4_OH (Sigma-Aldrich) in PBS, followed by washing in PBS.

### 3.14 Immunofluorescence

Both cellularized (with v-HCFs or a-HCFs) and decellularized samples were fixed in paraformaldehyde 4% in PBS (PFA, Alfa Aesar) for 15 min, washed with PBS, and cells were permeabilized with Triton X-100 (Sigma-Aldrich) 0.5% in PBS for 10 min. Samples were then blocked with bovine serum albumin (BSA, Sigma-Aldrich) 2% in PBS for 30 min, followed by staining with Phalloidin-Rhodamine (ThermoFisher) or primary and secondary antibodies, diluted in BSA 2% in PBS. Primary antibodies for fibroblasts staining were: Anti-Actin Smooth Muscle (α-SMA, Sigma Aldrich) and Anti-Discoidin Domain Receptor 2 (DDR2, ThermoFisher). Primary antibodies used for extracellular matrix protein detection were anti-Collagen I, anti-Collagen III, anti-Fibronectin, anti-Laminin, anti-Tenascin, (all purchased from Sigma-Aldrich), and anti-Collagen IV (Abcam). Secondary antibodies used were anti-mouse Alexa Fluor 555 and anti-rabbit Alexa Fluor 488 (both from ThermoFisher). Nuclei were counterstained with DAPI (Sigma-Aldrich). Samples were maintained in PBS during imaging by using Nikon Ti2-E fluorescence microscope (Nikon Instruments). Immunofluorescence experiments were performed in biological triplicate.

### 3.15 Two-photon microscopy

Two-Photon Excitation Fluorescence (TPEF) microscopy technique was applied on cellularized (with v-HCFs only) and decellularized 2D and 3D scaffolds, prepared for immunofluorescence analysis, in order to characterize cell arrangement and matrix organization also in the inner layers of 3D structures. In detail, α-SMA and cell nuclei were stained by Alexa Fluor 555 and DAPI, respectively, while Collagen I-and Collagen IV in the ECM were stained by Alexa Fluor 555 and Alexa Fluor 488, respectively.

The microscopy setup used for the analysis has been discussed in detail in a previous publication (Mortati et al., 2020). Briefly, samples were excited with laser pulses of about 6 ps length and 76 MHz repetition rate, with a wavelength of 800 nm for DAPI staining and of 922 nm for Alexa Fluor 555 (α-SMA) staining. The excitation source was focused on the sample using a water immersion objective (LUMPLFLN 40XW NA = 0.8, W.D. = 3.3 mm, Olympus) and the TPEF signal was collected in epi-direction using an Olympus Fluoview FV300 scanning head and an upright Olympus BX51WI microscope. A dichroic mirror separated the emitted signal of the two fluorophores in two different channels. Each channel was equipped with optical filters that allowed a transmission window matched with the fluorophore emission spectra.

A set of several 3D images were collected and stitched together in a bigger 3D reconstruction. The voxel pitch was about 0.7 μm × 0.7 μm × 5 μm respectively in the X, Y, Z axis for the 2D scaffold and 3D scaffold. In order to get a wide picture of the scaffolds, the 3D images were collected following a spatial grid with a step of about 300 μm using a motorized sample stage equipped with two stepper motors (PI M-229.255). The 2D scaffold was imaged collecting 6 × 6 3D images; the 3D scaffold was imaged collecting 10 × 10 3D images. The 3D stitching process was done by a custom-made ImageJ plugin, while the 3D reconstruction was made using the 3Dviewer and the ClearVolume ImageJ plugins.

Close-up images were taken with a Two-Photon Microscope Nikon A1R MP + Upright equipped with a femtosecond pulsed laser Coherent Chameleon Discovery (∼100 fs pulse duration with 80 MHz repetition rate, tunable wavelength output 660–1320 nm). A 25X water dipping objective with numerical aperture (NA) 1.1 and working distance (WD) 2 mm was employed for focusing the excitation beam and for collecting the TPEF signal. The latter was directed by a dichroic mirror to a series of three high sensitivity GaAsP detectors (non-descanned detection, allowing fast image acquisition) equipped with different filtering schemes able to select proper spectral range, resulting in three separated, simultaneously acquired channels: blue channel (415 < λ < 485 nm), green channel (506 < λ < 594 nm) and red channel (604 < λ < 679 nm). Imaging overlay of the three channels and processing was performed by the operation software for the microscope.

Images were acquired (except where explicitly reported) at an excitation wavelength of 950 nm, with a typical field of view of 500 µm × 500 µm (1024 × 1024 px), meaning an effective sampling of 0.5 µm/px, while the Z-scan incremental step was 1 µm. Estimated lateral and vertical resolution gave rise to an effective voxel of 0.4 µm × 0.4 µm × 1.5 µm, meaning that the 3D volumes images have been slightly oversampled in the *Z* direction while a correct sampling scheme has been adopted for XY image reconstruction. In order to have a suitable signal to noise ratio, the effective average time per image was set between 4 and 8 s: effective sampling frequency for Z-scan was between 125 and 250 mHz.

### 3.16 Atomic force microscopy force spectroscopy analysis

Atomic Force Microscopy Force Spectroscopy method was used to characterize the mechanical properties of the scaffolds at sub-micrometer scale. The local Young’s moduli of the PCL and PCL/PolyDOPA/G scaffolds were measured using a NanoWizard II AFM (JPK Instruments). A spherical indenter was made using a tipless cantilever (TL-FM-20 by Nanosensors) and a tungsten sphere of about 10 µm diameter (357421-10G by Aldrich Chemistry) bounded together with an epoxy adhesive cured with UV light. Thermal noise and Sader based method was used to obtain the cantilever spring constant (Sader et al., 2012) that was about 5.55 N/m, while the resonance frequency was about 66.5 kHz and the sensitivity about 33.2 nm/V. Elastic modulus has been measured in contact mode over a grid of 4 points in a square with a side length of 10 µm in correspondence of one of the scaffold rows and for each point the measure has been repeated fifty times, collecting 200 curves per measured area. These measurements have been repeated twice on two different areas of the same sample and in each measurement session it has been set ten different tip forces over the sample during extend segment with a value ranging from 30 nN to 210 nN and a step of 20 nN between each of them, collecting an overall of 4000 curves for each sample. The AFM piezo was set to move the tip over an extended distance of 6 µm in 500 ms. The local Young’s modulus of the scaffolds has been extracted using the Hertz’s spherical punch model over the extend curves (Sneddon, 1965). The measurements have been performed both in air and in water for both scaffold types. The same measurement procedure has been applied to the 2D PCL and 2D PCL/PolyDOPA/G samples both in air and water.

### 3.17 Statistical analysis

All experiments were performed in triplicates and data were presented as mean ± SD from three independent experiments. Data were analyzed with GraphPad Prism version 9.0 for Windows (GraphPad Software, www.graphpad.com), using two-way ANOVA analysis to compare results.

## 4 Results

The aim of this study was the *in vitro* engineering of human cardiac fibrotic tissue at early post-infarct stage, which is characterized by random tissue architecture (Talman and Ruskoaho, 2016a), stiffening respect to healthy cardiac tissue (Yang et al., 2002; Czubryt, 2012; van Nieuwenhoven and Turner, 2013), presence of specific cardiac extracellular matrix proteins (fibronectin, laminin, collagen I, III, IV and tenascin) (Castaldo et al., 2013), and a cell population mainly consisting of activated cardiac fibroblasts (MyoFs) (Yang et al., 2002; Czubryt, 2012; van Nieuwenhoven and Turner, 2013). 2D and 3D “bioartificial” scaffolds were designed and then cultured with v-HCFs from cardiac ventricular samples for 3 weeks, to depose their ECM and reproduce early post-infarct cardiac fibrotic tissue with different thicknesses. Additionally, a-HCFs isolated from atrial cardiac samples were also cultured on 2D and 3D scaffolds to evaluate the possibility to exploit the same scaffold platforms to engineer *in vitro* atrial fibrosis.

### 4.1 Scaffold morphological analysis

**FIGURE 1:**
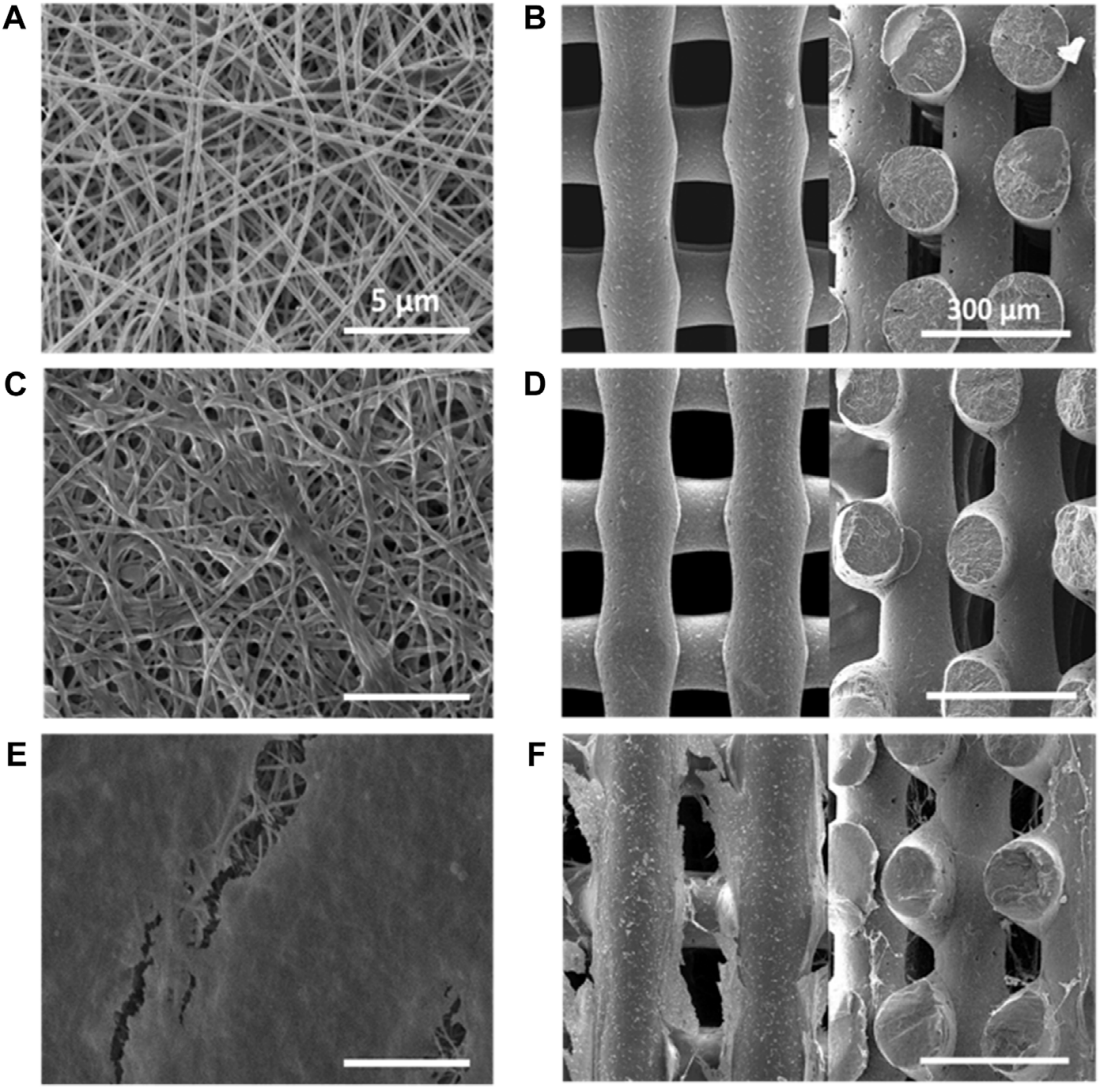
SEM images of PCL scaffolds: **(A)** 2D scaffold (top view); **(B)** 3D scaffold (left image: top view in x-y plane; right image: section in x-z plane); PCL/PolyDOPA/G scaffolds: **(C)** 2D scaffold (top view); **(D)** 3D scaffold (left image: top view in x-y plane; right image: section in x-z plane; decellularized PCL/PolyDOPA/G scaffolds after 21 days v-HCFs culture: **(E)** 2D scaffold (top view); **(F)** 3D (left image: top view in x-y plane; right image: section in x-z plane). Section images were acquired on 3D scaffold samples fractured in liquid nitrogen. Scale bars: 5 µm for 2D scaffolds **(A,C,E)**; 300 µm for 3D scaffolds **(B,D,F)**.

The electrospinning process parameters were optimized (applied voltage: 15 kV; solution flow rate: 0.25 mL/h; needle-collector distance: 13 cm) allowing the preparation of 2D PCL scaffolds with 60 µm average thickness. SEM images of 2D scaffolds evidenced the presence of randomly distributed nanofibers, free of defects (Figure 1A). Fiber size was in the 70–220 nm range, with an average size of 127 ± 33 nm (Supplementary Figure S1A), whereas pores showed an uniform distribution with 90% of pore diameters in the 0.5–1 µm range (Supplementary Figure S1B).

3D PCL scaffolds with square meshed geometry were fabricated by MEAM with 150 µm mesh sizes: (Supplementary Figure S2A). Supplementary Table S1 collects 3D scaffold geometrical characteristics in terms of pore and filament sizes. In 3D scaffolds filaments showed 135 ± 4 µm size. SEM images of 3D scaffold x-y and x-z sections confirmed the presence of interconnected pores (Figure 1B). The interpenetration of filaments belonging to overlapped layers was of around 30 µm. (as shown in Figure 1B and Supplementary Figure S2B). Theoretical porosity degree was evaluated considering the void space left from the infill density set by CAD file (30%), while measured porosity degree was calculated by a gravimetric method (46 ± 6%) (Supplementary Table S2).

The exposed surface area per scaffold was also calculated (Supplementary Table S3, Supplementary Figure S3), resulting 0.9 cm^2^ and 0.23 cm^2^ for 2D and 3D scaffolds, respectively.

### 4.2 PolyDOPA/G coating

#### 4.2.1 Coating efficiency and stability

**FIGURE 2:**
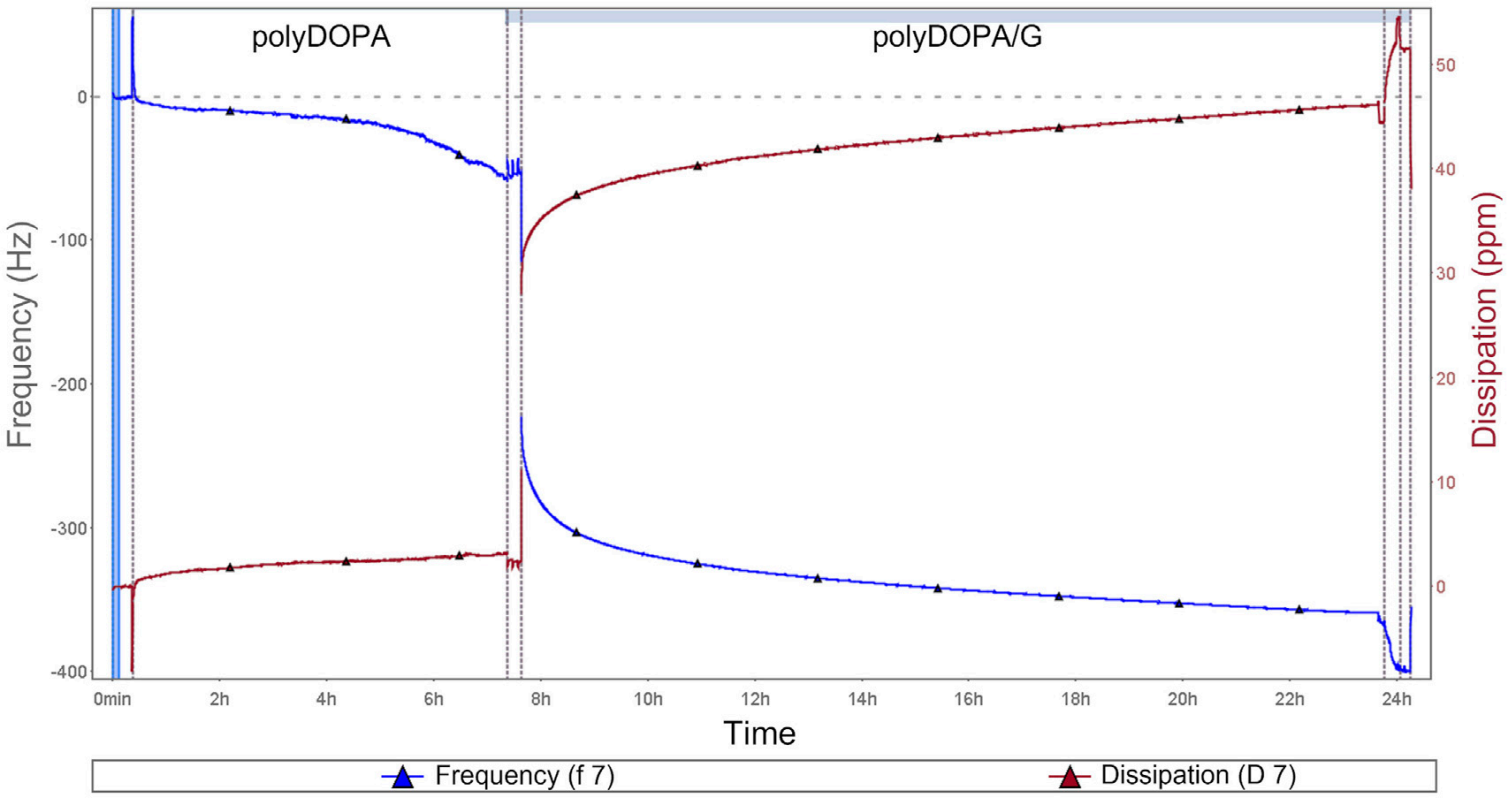
QCM-D analysis during polyDOPA/G deposition: blue curve refers to frequency, while red curve represents dissipation as a function of incubation time. All the phases of functionalization are displayed, including initial sensor preconditioning as well as washing steps after polyDOPA coating before G deposition.

2D and 3D scaffolds were surface functionalized with polyDOPA, as an intermediate layer for subsequent G grafting. Experimental parameters for polyDOPA functionalization of PCL scaffolds were derived from a previous study on poly (lactic acid-co-glycolic acid) PLGA surface functionalization, published by the same authors (Carmagnola et al., 2020). Initially, QCM-D analysis was performed to study polyDOPA/G coating efficiency applying previously selected parameters (Carmagnola et al., 2020). Figure 2 shows frequency and dissipation curves during sequential polyDOPA and G depositions on QCM-D gold sensor. Frequency decreased from 0 to −372 Hz as indicative of effective polyDOPA and polyDOPA/G mass deposition on the gold sensor. In detail, during the first 7 h, a gradual frequency decrease down to −58 Hz was observed, due to DOPA oxidation and self-polymerization of oxidized DOPA on the sensor resulting in polyDOPA coating. Giving the rigid nature of polyDOPA, during its deposition, dissipation changes were negligible. On the other hand, during subsequent G deposition, frequency steeply decreased, while dissipation increased as a function of incubation time, due to G low stiffness. After 16 h incubation in G solution of the polyDOPA coated sensor, frequency reached −372 Hz and dissipation increased up to 47 ppm. Using “QSense Dfind” software, layer thickness and deposited mass were evaluated. Sauerbrey model (based on Sauerbrey equation for rigid coating layers) was applied to estimate polyDOPA layer mass and thickness (Supplementary Figures S4A,C), while “Smartfit” method (suitable for modelling soft coating layers) was used to derive the same parameters for G coating (Supplementary Figures S4B,D). Comparison between estimation made using these two different methods is shown in Supplementary Figure S5. Deposited mass was estimated to be 1 μg/cm^2^ for polyDOPA layer and 7.4 μg/cm^2^ for subsequent G layer. PolyDOPA and G layers coating thicknesses were measured to be 10 nm and 74 nm, respectively. Hence, QCM-D analysis demonstrated the effective deposition of polyDOPA/G on gold sensor at the tested conditions, therefore such experimental parameters were further exploited for surface functionalization of PCL scaffolds.

**FIGURE 3:**
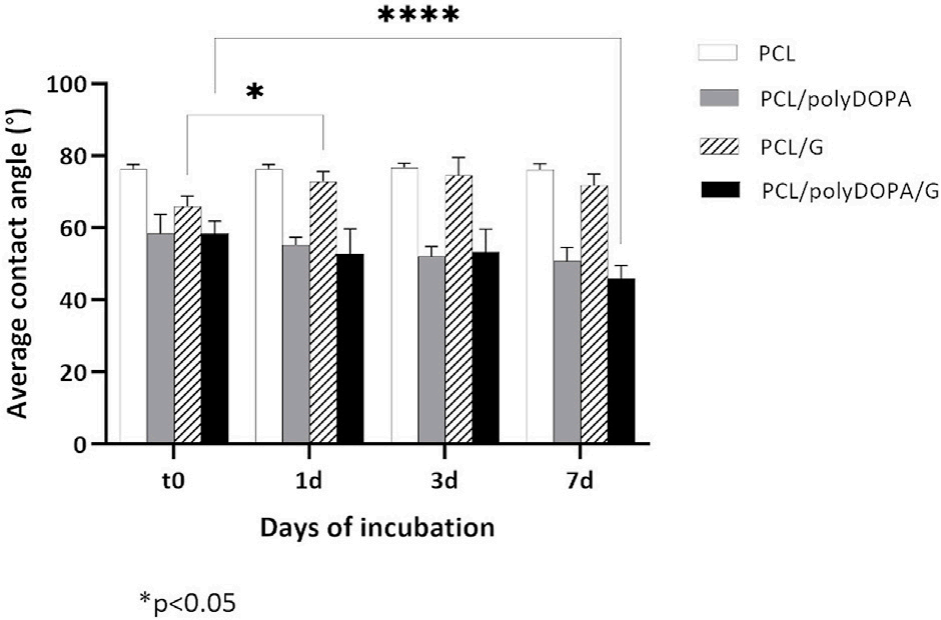
Average static contact angle values measured after 0, 1, 3, and 7 days (t0, 1d, 3d, and 7d) incubation in PBS at 37°C for PCL, PCL/polyDOPA, PCL/G and PCL/polyDOPA/G model films. Reported data are the average values ± standard deviation (*n* = 9). * *p*-value <0.05.

Static water contact angle analysis was performed to provide indirect evidence of polyDOPA/G coating effectiveness as well as coating stability during incubation in PBS (Table 1, Figure 3).

PCL films showed a static contact angle of 76° ± 1°, which decreased to around 58° after both polyDOPA and polyDOPA/G surface functionalization (Table 1). Control PCL films incubated in PBS up to 7 days maintained an average contact angle of around 76° (Figure 3). Static contact angle of PCL/polyDOPA films slightly decreased from 58° ± 5° to 51° ± 3° during incubation in PBS, but changes were not significant (Figure 3). On the contrary, PCL/polyDOPA/G films did not show significant variations in the static contact angle after up to 3 days incubation in PBS, while a small but significant decrease in the static contact angle value was measured after 7 days incubation in PBS (46° ± 4°) (Figure 3). Static contact angle of PCL/G samples, used as control, was higher than the other functionalized samples (66° ± 3°) and increased during incubation in PBS, reaching a contact angle value comparable with that of PCL film after 1 day (73° ± 3°), suggesting G release in the absence of polyDOPA intermediate coating (Figure 3).

#### 4.2.2 Scaffold surface functionalization

Morphological analysis of functionalized 2D and 3D PCL/PolyDOPA/G scaffolds was carried out by SEM (Figures 1C,D). Electrospun scaffolds preserved their nanostructure after surface modification. However, their average fiber diameter increased from 127 ± 33 nm (PCL) to 270 ± 70 nm (PCL/polyDOPA/G) and, consequently, entanglements between fibers also increased causing a slight reduction of pore area (Figure 1C). On the other hand, in 3D scaffolds no significant changes in filament surface morphology were observed after polyDOPA/G grafting (Figure 1D) respect to polyDOPA coated scaffolds (Figure 1B).

Wettability of unmodified and surface modified 2D scaffolds and 3D scaffolds was analyzed by sessile drop method, compared to film samples (Table 1, Figure 3). Static contact angle values of PCL 2D (141° ± 5°) and 3D (133° ± 3°) scaffolds were higher compared to PCL films (76° ± 1°), suggesting an influence of scaffold micro- and nanostructure on surface wettability. PolyDOPA coating increased scaffold surface wettability: 2D PCL/polyDOPA scaffolds showed an average static contact angle of 48° ± 8° which further reduced to 40° ± 6° for 2D PCL/polyDOPA/G scaffolds. However, for 3D PCL/polyDOPA and PCL/polyDOPA/G scaffolds, measurement of static contact angle was not possible due to the high surface wettability of samples (as demonstrated by the static contact angle values of films with the same composition, Table 1) and the presence of large pores.

BCA colorimetric assay showed that polyDOPA coating enhanced G functionalization. G amount per scaffold was initially calculated and then referred to the unit scaffold surface area, previously estimated and collected in Supplementary Table S3 (Table 2). For each scaffold type, G density significantly increased in the presence of polyDOPA pre-coating. As surface area of 2D scaffolds was higher than for 3D scaffolds (Supplementary Table S3), G amount per scaffold was significantly higher on 2D compared to 3D scaffolds, especially when polyDOPA pre-coating was present.

**TABLE 2:** Gelatin quantification through BCA colorimetric assay for 2D and 3D PCL/PolyDOPA/G scaffolds compared to PCL/G scaffolds.

Samples	G amount per scaffold (µg/scaffold)	G amount per estimated scaffold surface area^a^ (µg/cm^2^)
2D PCL/G	147 ± 36	163 ± 40
2D PCL/polyDOPA/G	532 ± 160	591 ± 178
3D PCL/G	30 ± 1	130 ± 4
3D PCL/polyDOPA/G	67 ± 9	291 ± 39

^a^ The estimated exposed surface area of each scaffold is collected in Supplementary Table S3.

**FIGURE 4:**
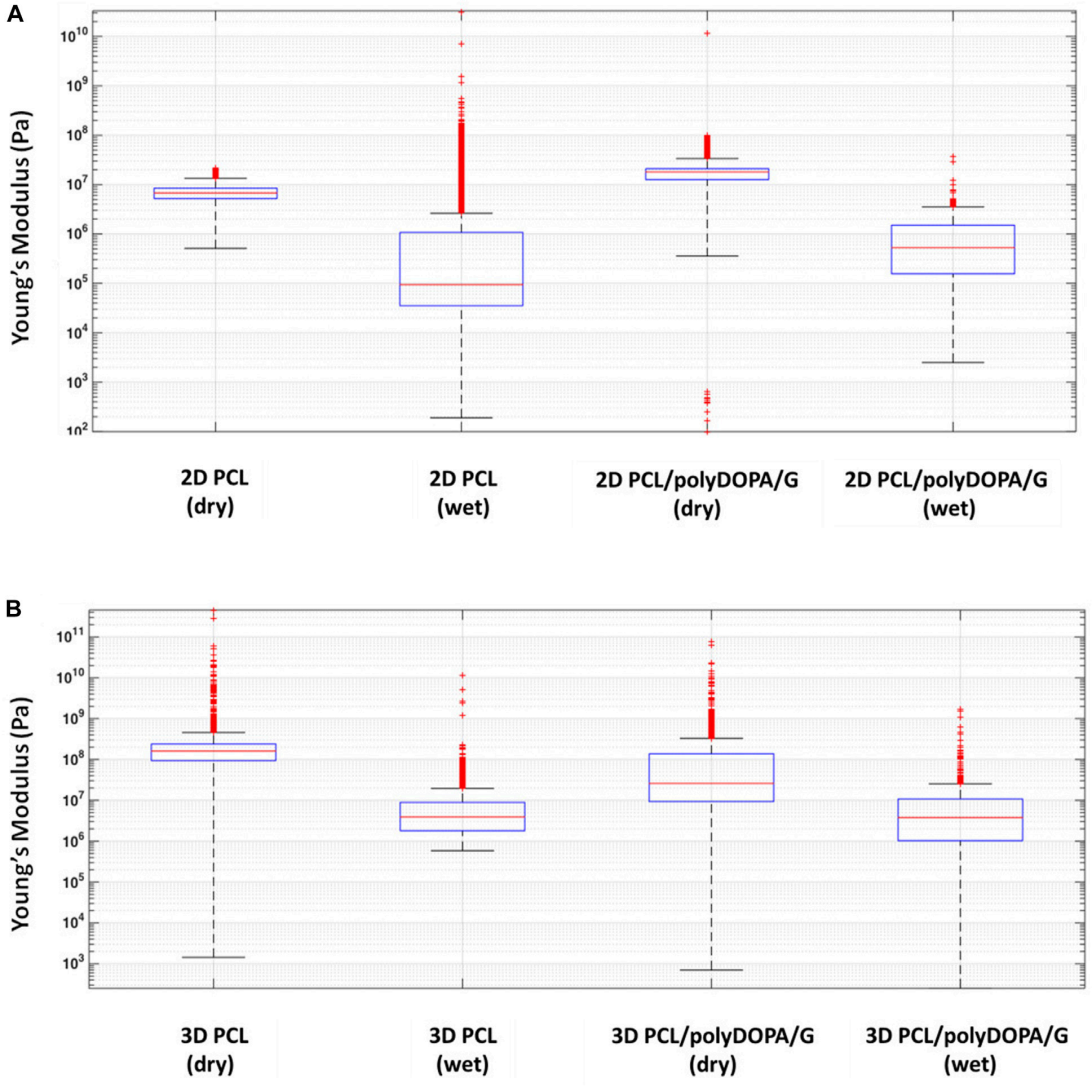
Boxplot graph of **(A)** the extracted Young’s modulus from the collected extended curves of 2D PCL and 2D PCL/polyDOPA/G samples in air and water immersion conditions and **(B)** 3D PCL and 3D PCL/polyDOPA/G samples in the same conditions.

AFM mechanical characterization was performed on 2D and 3D PCL and PCL/polyDOPA/G scaffolds in dry and wet conditions (Figure 4). For each scaffold type, the presence of the coating did not significantly change Young’s modulus. On the other hand, scaffold morphology was found to significantly affect surface mechanical properties, with 3D scaffolds showing around 10-fold higher stiffness than 2D scaffolds (Figure 4). Furthermore Young’s modulus decreased for both PCL and PCL/polyDOPA/G scaffolds in wet respect to dry testing conditions (Figure 4). In detail, according to boxplot average values, Young’s modulus decreased from 100 MPa to 10 MPa for 3D PCL and PCL/polyDOPA/G scaffolds (Figure 4B) and from 10 MPa to hundreds kPa for 2D PCL and PCL/polyDOPA/G scaffolds (Figure 4A) from dry to wet conditions.

### 4.3 *In vitro* cell cultures

#### 4.3.1 vHCFs viability on 2D and 3D scaffolds

v-HCFs were initially cultured on PCL and PCL/polyDOPA/G scaffolds and cell viability and cytotoxicity were evaluated after 1 and 7 days culture time, in order to confirm that PolyDOPA/G coating on PCL scaffolds could sustain initial cell adhesion and proliferation of the cells.

**FIGURE 5:**
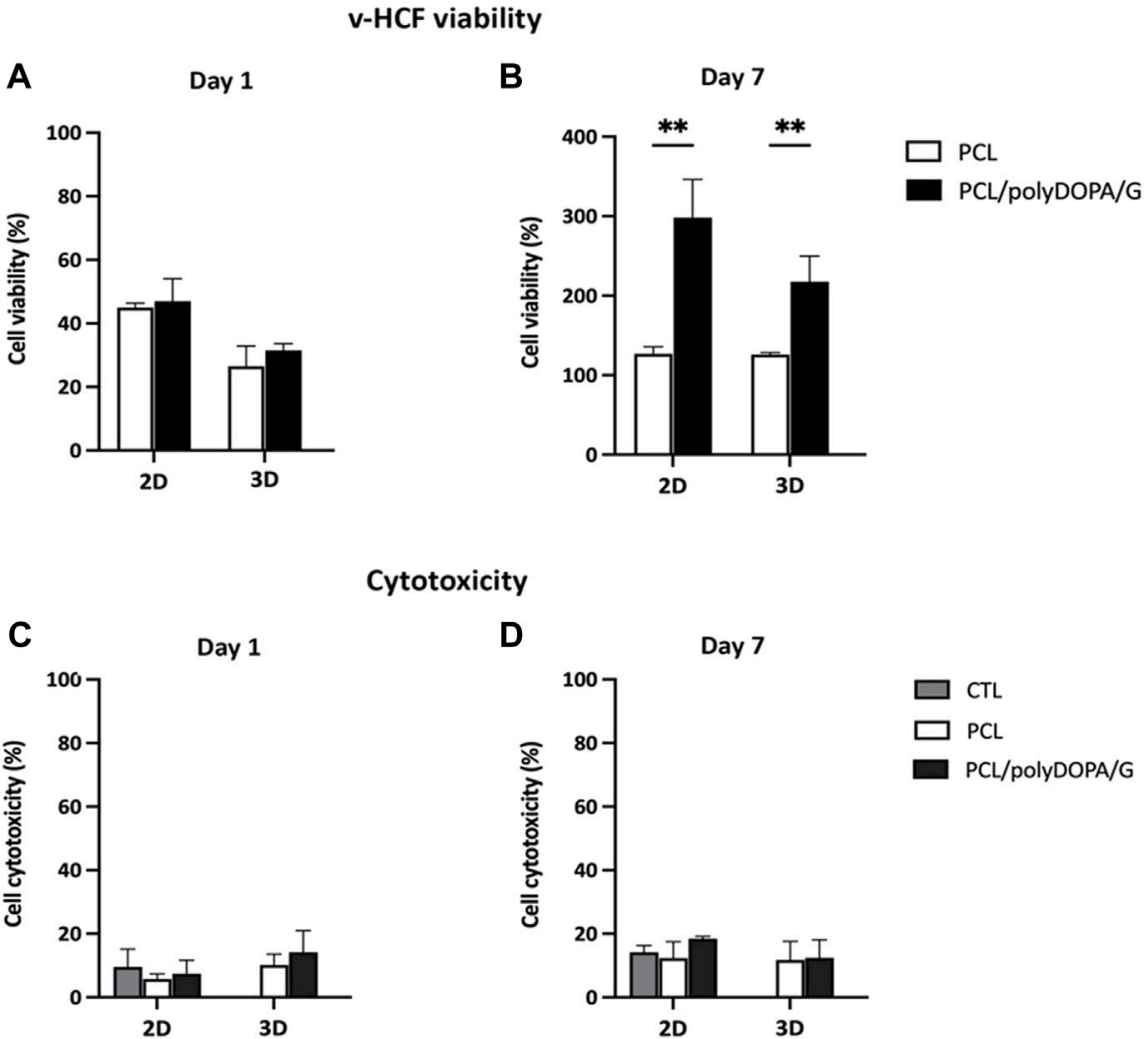
Cell viability percentage after 1 day **(A)** and 7 days **(B)** culture time on PCL and PCL/polyDOPA/G scaffolds. Percentage is calculated respect to viability of control cells cultured on gelatin-coated glass slides for the same time (1 and 7 days). Cytotoxicity percentage after 1 day **(C)** and 7 days **(D)** culture time on PCL and PCL/polyDOPA/G scaffolds. Percentage refers to 100% Cell Lysis control of cells cultured on gelatin-coated glass and treated with Lysis buffer (9% Triton X-100 in water). For all figures ** *p*-value <0.001.

Figure 5A shows cell viability percentage on the different scaffold types normalized respect to positive control (v-HCFs on gelatin-coated glass slide) as a function of culture time. After 1 day, cell viability on each scaffold type was lower than on the control: 47% for 2D PCL/polyDOPA/G and 26.5% for 3D PCL scaffolds. This result was due to lower cell seeding efficiency on scaffolds respect to flat culture plates. On the other hand, cell viability on 2D and 3D scaffolds was not affected by G functionalization after 1 day culture time (Figure 5A).

Cell viability significantly increased from day 1 to day 7 on all scaffold samples (Figure 5B). After 7 days, cell viability was significantly higher on 2D PCL/polyDOPA/G scaffolds (298%) compared to 2D PCL scaffolds (127%) (Figure 5B) and on 3D PCL/polyDOPA/G scaffolds (218%) compared to 3D PCL scaffolds (126%), respectively.

Cytotoxicity assays at the same time points (Figures 5C,D) showed no toxic effects on cells (<20% cytotoxicity for all conditions), and no significant differences between control and scaffolds.

#### 4.3.2 Human cardiac fibroblasts phenotype and morphology

##### 4.3.2.1 Long-term *in vitro* culture of human cardiac fibroblasts

**FIGURE 6:**
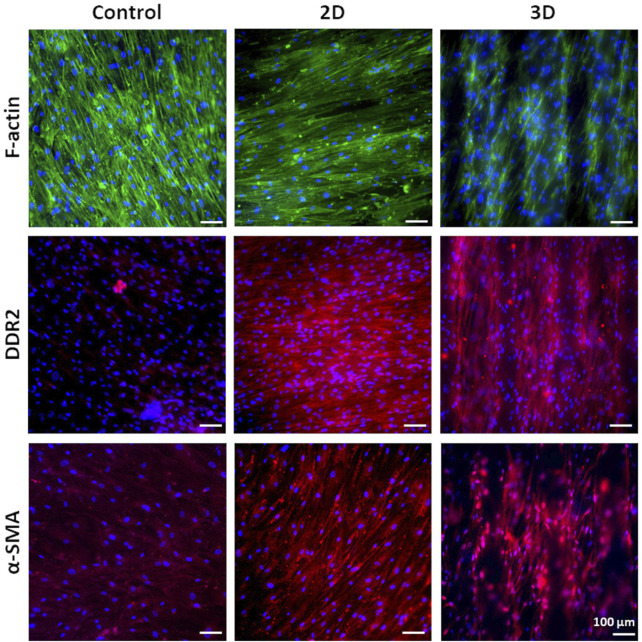
Phalloidin staining for F-actin and immunofluorescence analysis for DDR2 and α-SMA on v-HCFs cultured for 3 weeks on PCL/polyDOPA/G scaffolds and G-coated glass samples (control conditions). Cell nuclei were counterstained in blue with DAPI.

G is an adhesion protein favoring cardiac fibroblast attachment, proliferation, and biomatrix deposition at long culture time (3 weeks), as previously demonstrated by Castaldo et al. (2013). Hence, G was grafted on 2D and 3D PCL-based scaffolds with the aim to support long-term culture of v-HCFs and fibrotic tissue development following scaffold architecture. To the purpose, v-HCFs were cultured for 3 weeks on 2D and 3D PCL/polyDOPA/G scaffolds. Cells distribution on the scaffolds and their phenotype were studied by phalloidin staining of F-actin (a microfilament of the cytoskeleton) and immunofluorescence analysis of α-SMA (a specific marker for MyoFs) and Discoidin Domain Receptor 2 (DDR2) (a surface receptor of cardiac fibroblasts binding to collagen extracellular matrix filaments) (Figure 6). Phalloidin staining of F-actin highlighted cell coverage in all scaffolds and control samples after 3 weeks of culture. Also in the case of 3D scaffolds, v-HCFs could populate and bridge the pore area.

v-HCFs on 2D and 3D PCL/polyDOPA/G scaffolds highly expressed both DDR2 and α-SMA. On the contrary, v-HCFs on control samples only weakly expressed α-SMA and DDR2. Overall, such findings suggested that 2D and 3D PCL/polyDOPA/G scaffolds are suitable culture substrates for v-HCFs inducing their adhesion, proliferation and activation into MyoFs.

**FIGURE 7:**
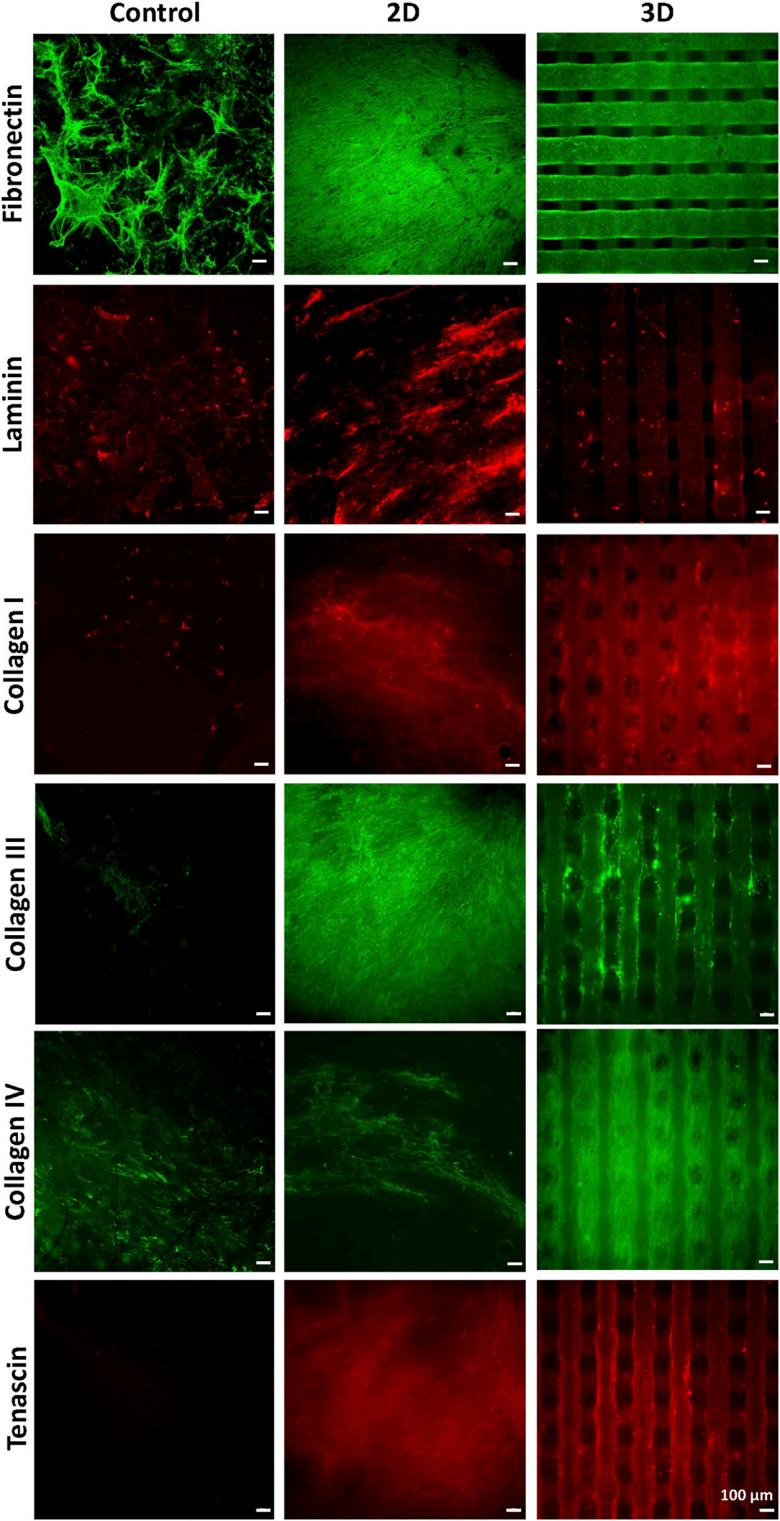
Immunostaining for Collagen -I, -III, -IV, for Fibronectin, Laminin, and Tenascin performed after decellularization, on PCL/polyDOPA/G scaffolds and control samples cultured for 3 weeks with v-HCFs.

Then, scaffolds were decellularized and their decoration with cell-secreted ECM was analyzed by SEM (Figures 1E,F) and immunofluorescence analysis of the typical proteins of cardiac pathological ECM, such as Fibronectin, Laminin, Collagen I, III and IV, and Tenascin (Figure 7) (Castaldo et al., 2013). SEM images showed that ECM uniformly coated 2D PCL/polyDOPA/G scaffolds (Figure 1E) and decorated the surface of external and the internal filaments of 3D PCL/polyDOPA/G scaffolds (Figure 1F). Immunofluorescence analysis evidenced that v-HCFs culture on scaffolds increased the deposition of ECM proteins compared to control samples (Figure 7). Although Fibronectin, Laminin, Collagen I, III and IV and Tenascin were detected on all the scaffold samples, their relative presence apparently varied depending on scaffold architecture, except for Fibronectin which was highly expressed on all samples. In detail, ECM on 2D scaffolds was mainly based on Fibronectin, Laminin, Collagen I, Collagen III and Tenascin. On the other hand, main ECM components present on 3D scaffolds were Fibronectin, Collagen I, Collagen IV and Tenascin. Limited amount of cardiac ECM was present on control samples, mainly composed by Fibronectin and Collagen IV. Interestingly, in agreement with results from immunofluorescence analysis of cultured v-HCFs, deposited ECM bridged scaffold pores in 3D scaffolds. Therefore, within 3 weeks culture on 2D and 3D PCL/polyDOPA/G scaffolds, v-HCFs built up pathological cardiac ECM on all scaffold types with complete ECM coverage, forming models of early-stage ventricular cardiac fibrotic tissue at different thicknesses.

Proof-of-concept results on the applicability of this approach to a-HCFs (isolated from atrial samples of patients with ischemic cardiomyopathy) was also performed with the aim to mimic early-stage atrial cardiac fibrotic tissue *in vitro*: immunostaining for F-actin, α-SMA, Fibronectin, Laminin, and Tenascin, was performed after decellularization, on 2D and 3D PCL/polyDOPA/G scaffolds cultured for 3 weeks with a-HCFs (Supplementary Figure S6).

#### 4.3.3 Advanced characterization of extracellular matrix and cell arrangement on scaffolds

Decellularized and cellularized 3D scaffolds were also analyzed using Two-Photon Excitation Fluorescence (TPEF) microscopy, to detect 3D cell and ECM arrangement on scaffolds. Indeed, fluorescence microscopy can fully inform on distribution of immunostained cells and ECM in 2D scaffolds, while in the case of 3D scaffolds inner scaffold structure cannot be explored through this technique.

**FIGURE 8:**
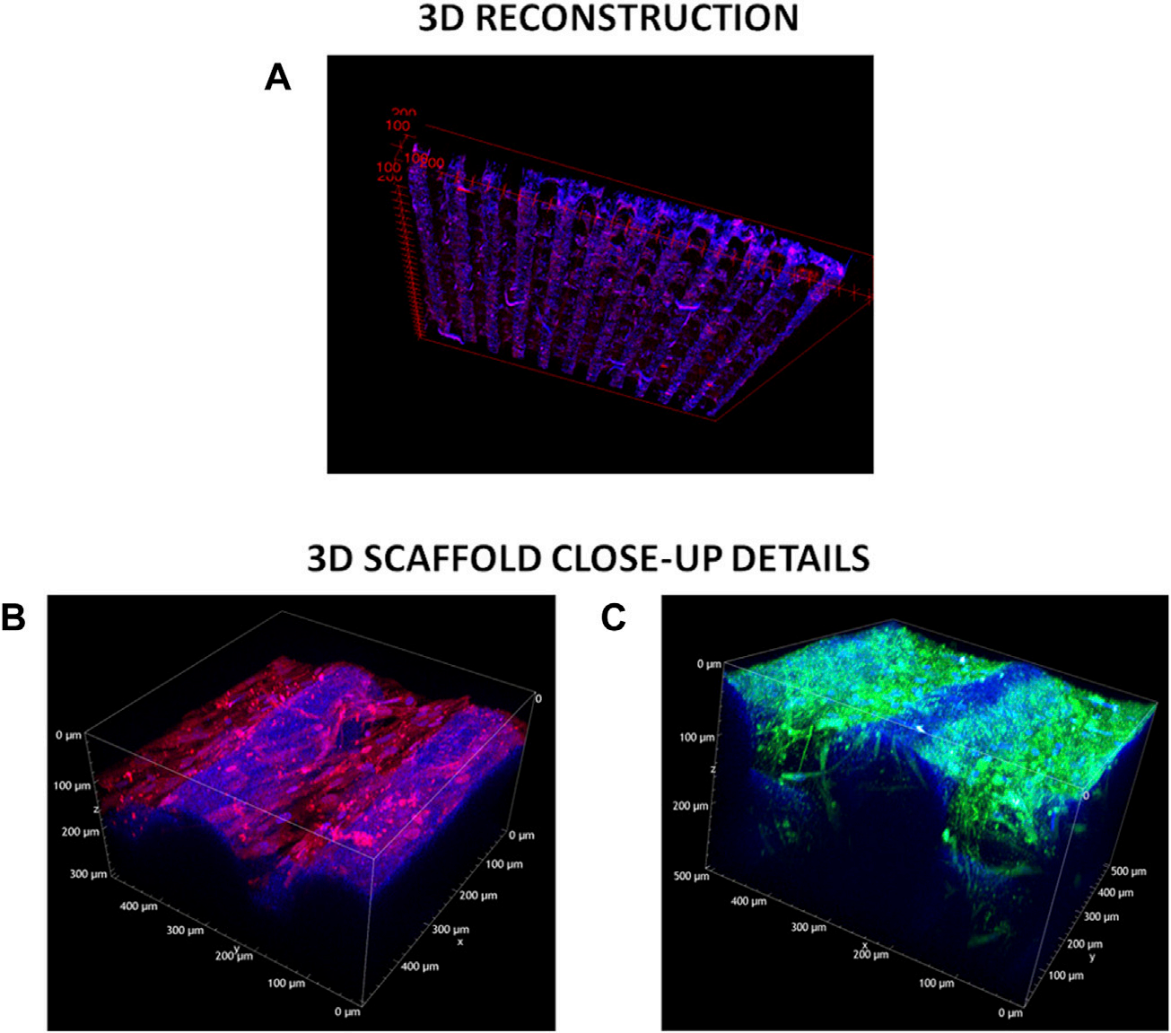
TPEF acquisitions. **(A)** 3D-cellularized scaffolds reconstruction. Blue refers to DAPI staining of cell nuclei, red indicates α-SMA—Alexa Fluor 555 immunostaining. **(B)** z-Stack measurement on the 3D cellularized scaffold stained for α-SMA. Excitation wavelength was 950 nm. Signal acquired in the red channel (641 +/− 37.5 nm) integrates the tail of the two-photon excited fluorescence response of Alexa Fluor 555. **(C)** z-Stack measurement on the 3D decellularized scaffolds stained for collagen I and IV. Excitation wavelength was 950 nm. Signal acquired in the green channel (550 +/− 44 nm) integrates the two-photon excited fluorescence response of both Alexa Fluor 488 and Alexa Fluor 555.

Tridimensional reconstructions of cellularized 3D scaffolds stained for α-SMA and cell nuclei (Figure 8A) showed that cells colonized the whole scaffold structures following their 3D geometry. Cells were also present at the filament junctions and on the inner filaments. More in detail, fluorescence signal from stained α-SMA was detected even from the bottom of the scaffold structure (limit of Z-depth ∼500 μm) (Figure 8B). The presence of elongated v-HCFs was evident in a supplementary movie that reconstructed the 3D volume in real-time (Supplementary Movies S1, S2).

The deposited extracellular matrix was imaged in Figure 8C: signal acquired in the green channel (550 +/− 44 nm) integrated the two-photon excited fluorescence response of both labeling fluorophores (Alexa Fluor 555 and Alexa Fluor 488), precluding the distinction of the two co-stained proteins (i.e., Collagen I and Collagen IV). The blue signal was due to a weak autofluorescence from both the PCL and residual DAPI stained-cell nuclei. Green fluorescent signal associated with Collagen I and Collagen IV deposition was detected both on the external surface and inner structure of 3D scaffolds (high value of Z-depth ∼300 μm). Furthermore, below the more external PCL filament, residual fibroblasts were observed. The presence of elongated residual fibroblasts was more evident from a supplementary movie that reconstructed the 3D volume in real-time (Supplementary Movie S2). The deposition of a dense ECM on scaffold filaments probably hindered complete v-HCFs removal during decellularization in static conditions.

For a comprehensive investigation, TPEF images were acquired also for 2D scaffolds, and are shown in Supplementary Figure S7.

## 5 Discussion

The wide incidence of cardiovascular diseases and the lack of regenerative therapies have increased the interest toward the design of *in vitro* models of human cardiac fibrotic tissue for the preclinical validation of new therapeutic approaches. Previously developed models of cardiac fibrotic tissue were mainly based on cellularized hydrogels (Sadeghi et al., 2017; Conant et al., 2017). Although they succeeded in mimicking pathological cardiac tissue more closely than 2D cell cultures, they could not replicate specific features of human cardiac fibrotic tissue, such as fibrosis type, stage and size (Zhao et al., 2014; Sadeghi et al., 2017). Novelty of this work arises from the design of 2D and 3D bioartificial scaffolds providing suitable structural and biochemical cues to cells for the *in vitro* engineering of early ‐stage post-infarct cardiac fibrotic tissue, mimicking its typical hallmarks, such as cardiac ECM composition, mechanical stiffness, lack of cell orientation and cell population mainly consisting of MyoFs (Yang et al., 2002; Czubryt, 2012; van Nieuwenhoven and Turner, 2013).

PCL was selected for scaffold fabrication and was processed through two different techniques. Electrospun 2D PCL scaffolds (∼60 µm thickness) showed random nanofibrous structure, an average fiber size of 127 ± 33 nm and pores with lower size than 1 μm, closely resembling ECM architecture of early-stage cardiac fibrotic tissue (Figure 1A) (Talman and Ruskoaho, 2016b; Keirouz et al., 2020). 3D PCL scaffolds were fabricated by MEAM with reproducible and controlled 150 µm square grid-shaped geometry and 7 layers (Figure 1B). This architecture was selected to avoid preferential orientation of cells during *in vitro* culture in order to reproduce the non-oriented cell arrangement of early-stage infarcted tissue (Castilho et al., 2017). Measured porosity degree of 3D scaffolds was ∼46%, ∼16% higher than the theoretical porosity value (Supplementary Table S2). The interpenetration of filaments belonging to overlapped layers was of around 30 μm, leading to stable scaffolds with interconnected porosity (as shown in Figure 1B and Supplementary Figure S2B). Interestingly, the different geometries of 2D and 3D scaffolds were also associated with significant differences in scaffold surface area (Supplementary Table S3), which then affected surface functionalization and cell adhesion.

PCL scaffolds were then functionalized with G, an adhesion protein supporting *in vitro* culture of v-HCFs, by a mussel-inspired method, previously developed by some of the authors (Carmagnola et al., 2020). Previous studies by Castaldo et al. have shown that the culture of cardiac fibroblasts isolated from atrial samples on G-coated culture plates for 3 weeks allowed the *in vitro* deposition of cardiac biomatrix with similar composition to cardiac ECM. Hence, long-term culture of cardiac fibroblasts on G-functionalized scaffolds was aimed at v-HCFs differentiation into MyoFs and deposition of cardiac ECM following 2D or 3D scaffold architecture. The progressive formation of polyDOPA/G coating on the gold sensor of QCM-D equipment was monitored, and measured frequency shifts allowed to estimate coating thickness (84 nm) (Figure 2). Based on the different stiffness of PolyDOPA and G, different models were applied to relate measured dissipation (*Δf*) to mass change (Figure 2, Supplementary Figures S4, S5). Particularly, “Sauerbrey” and “Smartfit” models were found to be appropriate to estimate polyDOPA and G coating thickness, respectively (Supplementary Figures S4, S5).

Due to the nanoscale thickness of the coating, its application on 3D scaffolds with micrometric filaments did not alter scaffold morphology (Figure 1B compared to Figure 1D), while the average fiber size of 2D scaffolds was significantly increased and their pore area reduced upon PolyDOPA/G coating application (Figure 1A compared to Figure 1C).

After PolyDOPA/G coating, surface hydrophilicity of substrates increased as assessed by static contact angle analysis of model PCL-based film samples (58° ± 3° vs. 76° ± 1° for PCL films, Table 1). However, static contact angle of substrates depends both on chemical nature of constituent materials and surface topography (geometry, roughness and presence of pores). In fact, nanostructured PCL 2D scaffolds have high surface hydrophobicity (Bartolo et al., 2006) that strongly decreased after polyDOPA/G coating (Table 1). Static contact angle of surface functionalized 3D scaffolds could not be measured due to their surface hydrophilicity and presence of relatively large pores (Table 1).

The presence of polyDOPA pre-coating allowed stable functionalization with higher G amount, compared to G physical adsorption (Table 2). Interestingly, G surface density on polyDOPA/G coated scaffolds was higher in 2D scaffolds due to their higher surface area (591 ± 178 μg/cm^2^ for 2D scaffolds vs. 291 ± 39 μg/cm^2^ for 3D scaffolds) (Table 2). Stability of polyDOPA/G coating in water-based medium and its high G density were advantageous for optimal support of *in vitro* cell culture on scaffolds. Cardiac tissue after MI shows patient-specific features depending on fibrosis size (thickness and width) and stage (early or late stage based on the different maturation/remodeling level of the cardiac scar) and is hallmarked by a range of Young’s modulus, from dozens of kPa to few MPa (Chelnokova et al., 2016; Sadeghi et al., 2017; Emig et al., 2021). Hence, scaffolds for cardiac tissue engineering have been previously designed to display a Young’s modulus varying from 20 kPa to 90 MPa (Nguyen-Truong et al., 2020). The mechanical properties of 2D and 3D PCL and PCL/polyDOPA/G scaffolds were herein determined through AFM technique both in dry and in wet conditions (Hiesinger et al., 2012; Nguyen-Truong et al., 2020), with the aim to investigate the mechanical properties sensed by cells. Experimental data obtained in wet condition for both scaffolds showed Young’s moduli in the range of scar tissue stiffness (Chelnokova et al., 2016) (hundreds kPa for 2D scaffolds and around 10 MPa for 3D scaffolds, as shown in Figure 4). Due to its nanometric thickness, polyDOPA/G coating did not alter scaffold surface mechanical properties, which only depended on scaffold structures. Indeed, 2D scaffolds were softer than 3D scaffolds due to their lower thickness (60 µm compared to around 800 µm for 3D scaffolds) and nanostructured architecture. Whereas polyDOPA/G coating did not affect surface mechanical properties, its role was that to support the adhesion of cardiac fibroblasts (Castaldo et al., 2013) for scaffold decoration with cardiac ECM. Furthermore, the high scaffold surface stiffness could trigger cardiac fibroblasts activation in their fibrotic phenotype (Porter and Turner, 2009) for the deposition of a pathological cardiac ECM on scaffolds to mimic cardiac fibrotic tissue. In detail, the developed 2D and 3D scaffolds were aimed at the design of patchy and compact fibrotic tissue (Rog-Zielinska et al., 2016) at different thicknesses, reproduced by using respectively 2D and 3D substrates with remarkable dimensional scale differences. As previously described, cardiac fibrotic tissue is characterized by morphological changes and stiffening due to cell deposition of ECM proteins such as collagen type I and type III (Porter and Turner, 2009; Castaldo et al., 2013; Lucena et al., 2014). Such remodeled tissue prevents ventricle wall rupture and triggers a persistent activation of cardiac fibroblasts. v-HCFs, isolated from human ventricle samples and representing the main cell population in post-infarct cardiac fibrotic tissue were cultured on scaffolds. After 1 day culture time, cell viability was lower compared to control samples, as a consequence of the limited seeding efficiency on 2D/3D porous scaffolds vs. 2D plain substrates (Figure 5A). This hypothesis was confirmed by the lack of cytotoxic effects by scaffolds (Figures 5C,D) and the significant increase of cell viability at 7 days, suggesting cell proliferation (Figure 5B). Cell viability at 7 days was higher in 2D than 3D scaffolds due to their lower pore size (enhancing cell seeding efficiency) and increased scaffold surface area and G surface density (favoring cell adhesion), respect to 3D scaffolds (Figure 5B).

Based on that, after assessing the ability of PCL/polyDOPA/G scaffolds to support v-HCFs adhesion and proliferation, cells were cultured on scaffolds for 3 weeks to allow ECM deposition. Immunofluorescence analysis evidenced high cellularization of scaffolds compared to control samples after 3 weeks (Figure 6). Cellularization was complete on 2D and 3D scaffolds, as also evidenced by reconstructed TPEF images (Figure 8). Analyzed markers of v-HCFs fibrotic phenotype were α-SMA and DDR2. Particularly, α-SMA is a widely recognized marker of myofibroblast differentiation in the heart (Baum and Duffy, 2011; Tarbit et al., 2019). Figure 6 shows its expression in v-HCFs cultured on scaffolds, while it was only weakly expressed by the same cells cultured on control G coated glass slides. This result highlighted the ability of scaffolds to provide well-defined extracellular biochemical, mechanical and topographical cues to v-HCFs, allowing the acquisition of the same fibrotic phenotype present in post-infarct cardiac tissue. On the other hand, DDR2 is a surface receptor found in both cardiac fibroblasts and myofibroblasts. However, its expression has been found to increase in fibrotic cardiac tissue (George et al., 2016). Hence, *in vitro* DDR2 expression could be used as benchmark to determine the level of fibrosis mimicked by *in vitro* models, through a comparison with DDR2 expression level in human cardiac fibrotic tissue samples with different severity degrees. Interestingly, DDR2 was highly expressed by v-HCFs cultured on scaffolds, while only weakly expressed by control cells.

The production of cellular ECM on scaffolds was analyzed by immunofluorescence and TPEF after their decellularization. It is well established that fibrotic cardiac tissue is characterized by high production of collagens (Sullivan et al., 2014; Perestrelo et al., 2021) and fibronectin (Jugdutt, 2003). Designed scaffolds demonstrated to support the deposition of collagens and fibronectin by cells (Figure 7). Interestingly, 2D scaffolds, having reduced pore size and increased surface area than 3D scaffolds, better supported the deposition of pathological cardiac ECM than 3D scaffolds. In the case of electrospun 2D scaffolds, this result was attributed to their closer biomimicry of the random nanofibrous structure of cardiac fibrotic ECM. This effect was also reflected by the different collagens expressed on 2D versus 3D scaffolds: 2D structure stimulated the deposition of higher amount of collagen III, which has been shown to increase its expression during the first week after myocardial infarction episode, with a switch towards collagen Type I after 4 weeks (Sullivan et al., 2014). On 3D scaffolds, v-HCFs, produced their ECM bridging pore gaps after 3 weeks culture time, as shown by both immunofluorescence and TPEF images (Figure 8). SEM analysis confirmed the successful deposition of cardiac ECM on scaffolds, forming a homogeneous monolayer on 2D scaffolds and decorating PCL-based filaments in the case of 3D scaffolds. TPEP analyses confirmed such findings (Supplementary Figure S7). TPEP and SEM analyses evidenced the successful culture of v-HCFs on the inner scaffold layers in the case of 3D scaffolds (Figures 1F, 8). On the other hand, on control substrates (glass slides with physically absorbed G), biomatrix composition did not resemble pathological cardiac ECM (Figure 7), probably due to the excessive stiffness of glass slides [65 GPa (Kudo and Kinoshita, 2014)] compared to post-infarct tissue. Indeed, excessively soft or hard substrates do not provide adequate mechano-transduction signaling to cells (Yeung et al., 2005). PCL/polyDOPA/G sample stiffness, ranging from hundreds kPa to 10 MPa provided proper signaling to v-HCFs, allowing the deposition of cardiac ECM on scaffolds.

Additionally, the same scaffolds were populated with a-HCFs, cardiac fibroblasts of atrial origin, with the aim to mimic atrial fibrotic tissue *in vitro*. After 3 weeks culture on scaffolds, a-HCFs of atrial origin expressed α-SMA and produced ECM rich in fibronectin and laminin, and with higher tenascin content in 2D respect to 3D scaffolds (Supplementary Figure S6). However, a-HCFs did not deposit collagen, which is a key marker of cardiac fibrotic tissue (Supplementary Figure S6). This result suggested a different behavior of a-HCFs and v-HCFs and the need for additional chemical or physical stimuli during a-HCFs cultures: for example, the addition of TGF-β to culture medium (Leask, 2010; Sadeghi et al., 2017) and/or hypoxic culture conditions could allow the deposition of a collagen-rich ECM decorating the scaffolds (Visone et al., 2021; Occhetta et al., 2018) for closer mimicking atrial fibrosis.

The designed scaffolds also represent culture platforms, which could be exploited to model different types of cardiac fibrosis, by tailoring *in vitro* culture conditions, as demonstrated by the attempt to model atrial fibrosis Through a-HCF culture on scaffolds.

One limitation in this study was the development of cardiac fibrosis models based on one cell type (human cardiac fibroblasts). The interaction between cardiac fibroblasts and other cells, such as cardiomyocytes, could be studied in the future by optimizing cell co-culturing conditions on scaffolds (Hussain et al., 2013). However, it is important to underline that the here developed models based on one cell type appear promising for *in vitro* investigation on the efficiency of direct reprogramming of cardiac fibroblasts into induced cardiomyocytes, in different fibrotic settings, without the interference of pre-existing cardiomyocytes (Lee et al., 2019).

Lastly, in this work early-stage post-infarct cardiac fibrotic tissues were engineered by v-HCFs culture on 2D and 3D PCL/PolyDOPA/G scaffolds through scaffold geometries avoiding preferential orientation of cells in one direction. In the future, the employment of oriented scaffolds based on the same materials could be exploited to mimic late-stage post-infarct cardiac fibrosis, which is characterized by tissue orientation.

As a conclusion, the availability of specific *in vitro* models of human cardiac fibrotic tissue is a novel concept which appears extremely important for preclinical tuning of therapies to treat patient-specific pathological conditions.

## 6 Conclusion

In this work, 2D and 3D bioartificial PCL/polyDOPA/G scaffolds were prepared, provided with biomimetic biochemical and biophysical properties respect to early-stage post-infarct cardiac fibrotic tissue, and able to support long-term culture of human cardiac fibroblasts, favoring their adhesion, proliferation, differentiation into myofibroblasts and deposition of pathological cardiac ECM. Particularly, 2D electrospun PCL/polyDOPA/G scaffolds supported the deposition of a compact pathological ECM layer upon long-term culture of v-HCFs, leading to 2D models of early-stage post-infarct cardiac fibrotic tissue. 3D scaffolds promoted complete cellularization and pathological cardiac ECM decoration of the whole scaffold structure, including pore bridging, leading to 3D models of early-stage post-infarct cardiac fibrotic tissue. Beside 2D and 3D post-infarct fibrosis models, long-term culture of a-HCFs on the same scaffold platforms could be useful to engineer *in vitro* 2D and 3D atrial fibrosis. However, additional physical and/or biochemical stimulations are required to trigger a-HCF deposition of a collagen-rich pathological cardiac ECM for a closer reproduction of atrial fibrosis.

Overall, results demonstrated that both 2D and 3D PCL/polyDOPA/G scaffolds were suitable substrates to support HCFs adhesion, proliferation, fibroblasts-to-myofibroblasts differentiation and deposition of pathological cardiac ECM by v-HCFs for the *in vitro* engineering of human cardiac fibrotic tissue at early post-infarct stage. Such models are promising to engineer human cardiac fibrotic tissue with patient-specific features, such as fibrosis type, extension, and stage, based on scaffold structure (architecture; width; thickness) and culture conditions (types of cultured cells; physical and biochemical stimulations) and may deserve future interest for the preclinical assessment of new therapies.

## Data Availability

The raw data supporting the conclusion of this article will be made available under request to the authors.
