# Supplementary material for: Biomimetic design of bioartificial scaffolds for the in vitro modelling of human cardiac fibrosis
Source: Front Bioeng Biotechnol. 2022 Nov 24;10:983872. doi: 10.3389/fbioe.2022.983872 (PMC9731288; doi:10.3389/fbioe.2022.983872)
Supplement: Supplementary file 1 [file DataSheet1.pdf]

# Biomimetic design of bioartificial scaffolds for the *in vitro* modelling of human cardiac fibrosis.

Mattia SPEDICATI [a, b, c, 1], Gerardina RUOCCO [a, b, c,1], Alice ZOSO [a, b, c,1], Leonardo MORTATI [e] Andrea LAPINI [d,e], Andrea DELLEDONNE[d], , Carla DIVIETO [e], Veronica ROMANO [f], Clotilde CASTALDO [f], Franca DI MEGLIO [f], Daria NURZYNSKA [g], Irene CARMAGNOLA [a, b, c], Valeria CHIONO [a, b, c]

[a] Department of Mechanical and Aerospace Engineering, Politecnico di Torino, Torino TO, Italy

[b] POLITO Biomedlab, Politecnico di Torino, Torino TO, Italy

[c] Interuniversity Center for the promotion of the 3Rs principles in teaching and research, Italy.

[d] Department of Chemistry, Life Science and Environmental Sustainability, University of Parma, Parma PR, Italy

[e] Istituto Nazionale di Ricerca Metrologica (INRIM), Torino TO, Italy

[f] Department of Public Health, University of Naples 'Federico II', Napoli NA, Italy

[g] Department of Medicine, Surgery and Dentistry "Scuola Medica Salernitana", University of Salerno, Salerno SA, Italy

[1]Co-first authors

Corresponding author: Valeria Chiono valeria.chiono@polito.it

**Keywords**: bioartificial, cardiac fibrosis, *in vitro* model, poly(caprolactone), gelatin, extracellular matrix

# Supplementary Material

## *Morphological characterization of 2D and 3D scaffolds*

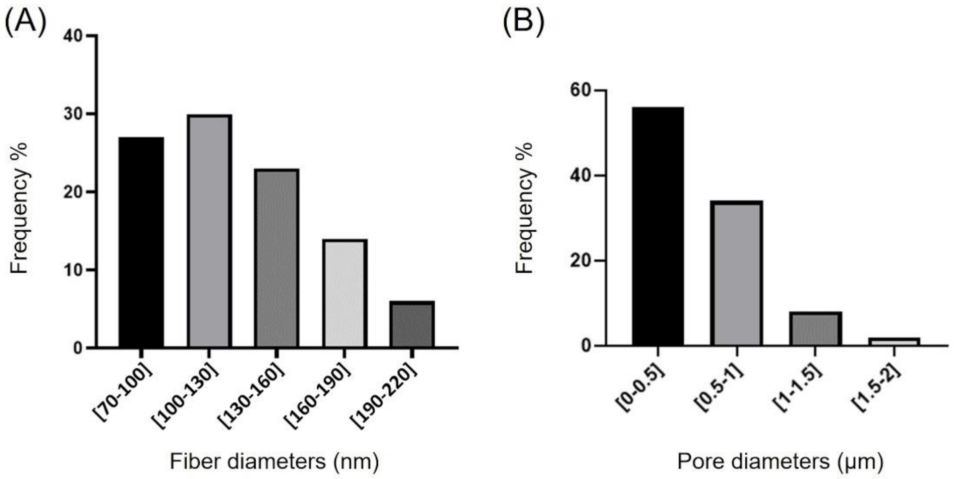


**Figure S1**. Percentage distribution of fiber diameters **(A)** and percentage distribution of pores size **(B)** for 2D scaffolds, measured through ImageJ analysis of SEM images of electrospun PCL membranes.

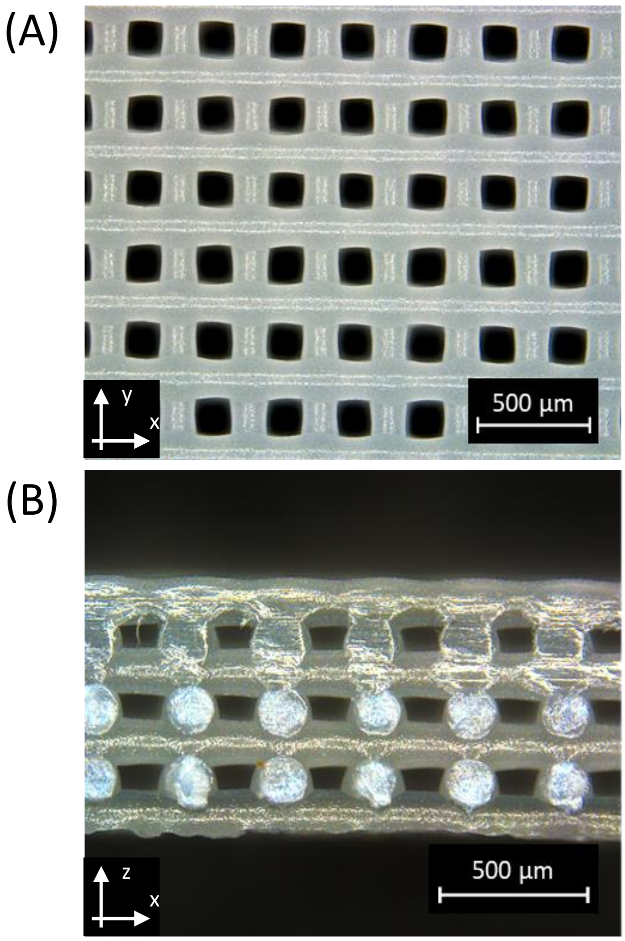


**Figure S2.** Optical microscope images of 3D scaffolds: **(A)** top surface and **(B)** section.

**Table S1.** Scaffold geometric characteristics were evaluated by analyzing optical microscope images by ImageJ software. Each parameter was expressed as mean value ± standard deviation. Pore size on surface (x-y plane, Figure S2A) and in section (x-z plane, Figure S2B) was evaluated by measuring width of empty space between filaments.

| **x-z plane** | | **x-y plane** | |
|---|---|---|---|
| Filament spacing z (μm) | Filament spacing x (μm) | Filament size (μm) | Filament spacing x, y (μm) |
| 75 ± 4 | 148 ± 6 | 135 ± 4 | 148 ± 6 |

**Table S2**. Theoretical porosity (calculated from Infill Density data, Equation 2) and measured porosity (obtained by gravimetric method, Equation 1) for 3D scaffolds.

| **Theoretical porosity (%)** | **Measured porosity (%)** |
|---|---|
| 30 | 46 ± 6 |

**Table S3**. Exposed surface area for 2D scaffolds (disk shape, 12 mm diameter, 0.06 mm thickness) and 3D scaffolds (square shape, 7×7 $mm^2$, 0.8 mm thickness).

| | **2D** | **3D** |
|---|---|---|
| Exposed surface ($cm^2$) | 0.9 | 0.23 |

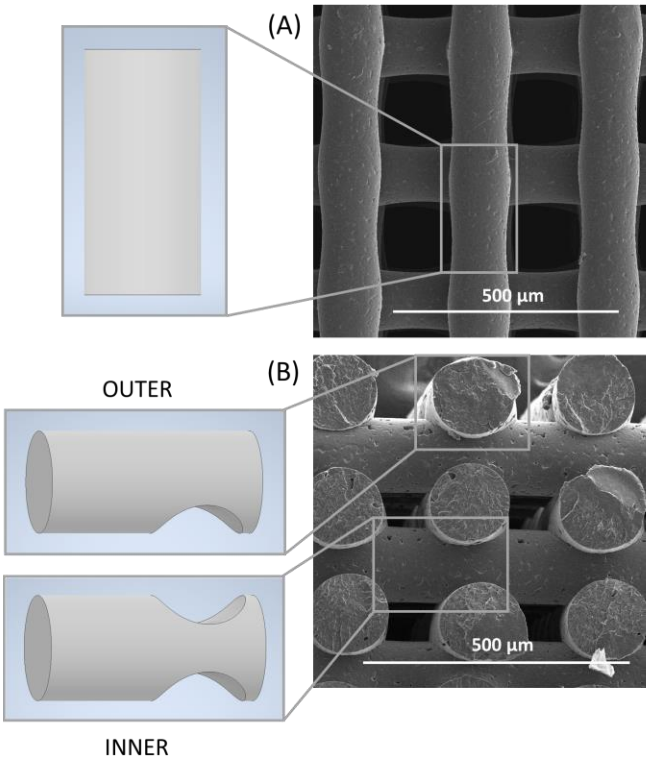


**Figure S3**. CAD model used to calculate exposed surface area for 3D scaffolds (**(A)** surface, **(B)** section). Magnified images show how crossed filaments were modelled. Surface of filaments present in the top layer (outer) was calculated considering their crossing with below filaments, while surface of filaments in the bottom layer was the half respect to the surface calculated for the filaments in inner layers.

### *G coating characterizations*

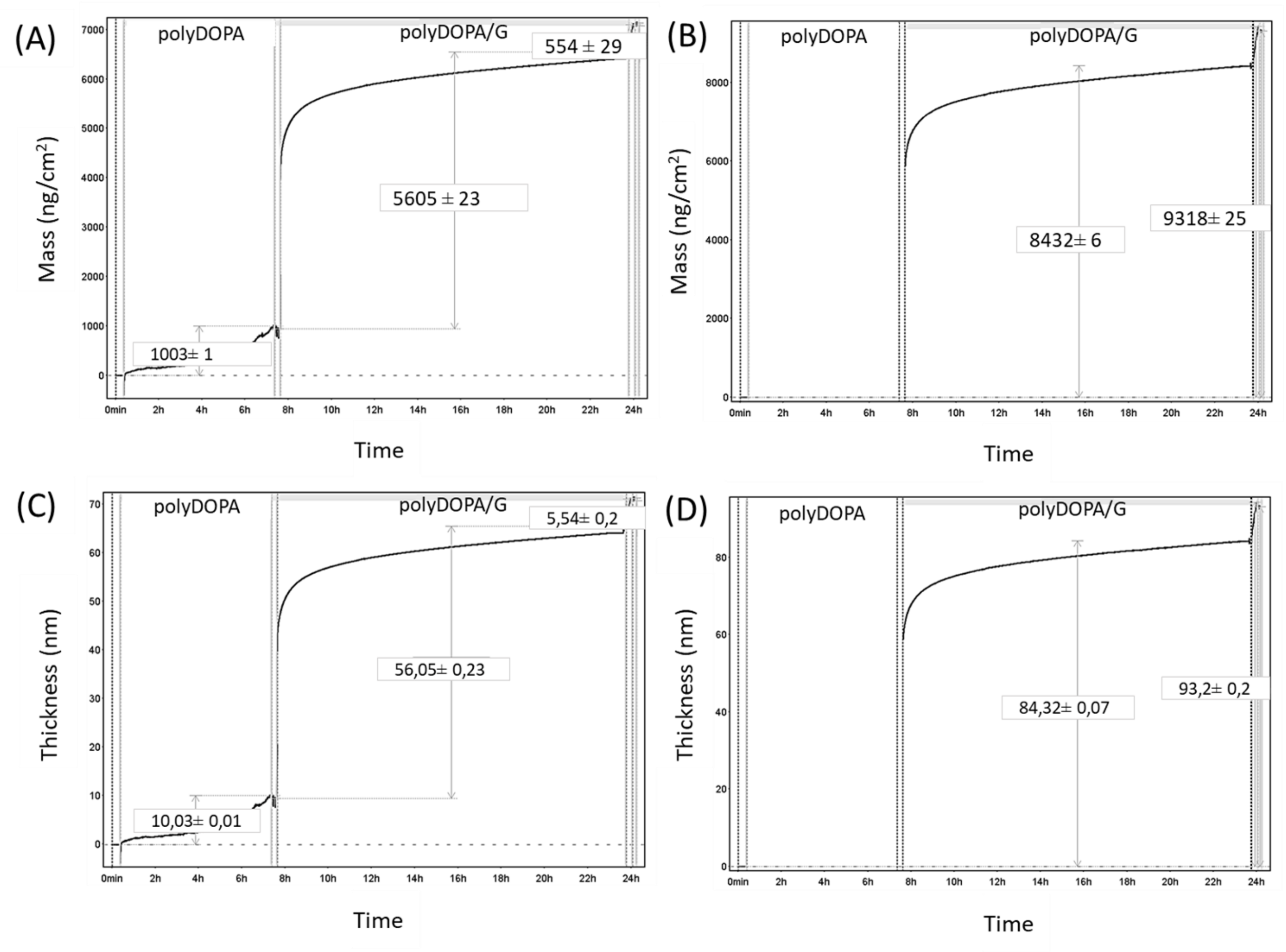


**Figure S4.** Mass **(A, B)** and thickness **(C,D)** of the coating layer as a function of incubation time, as evaluated by QCM-D analysis exploiting the Sauerbrey model **(A,C)** and "Smartfit" method **(B,D)**.

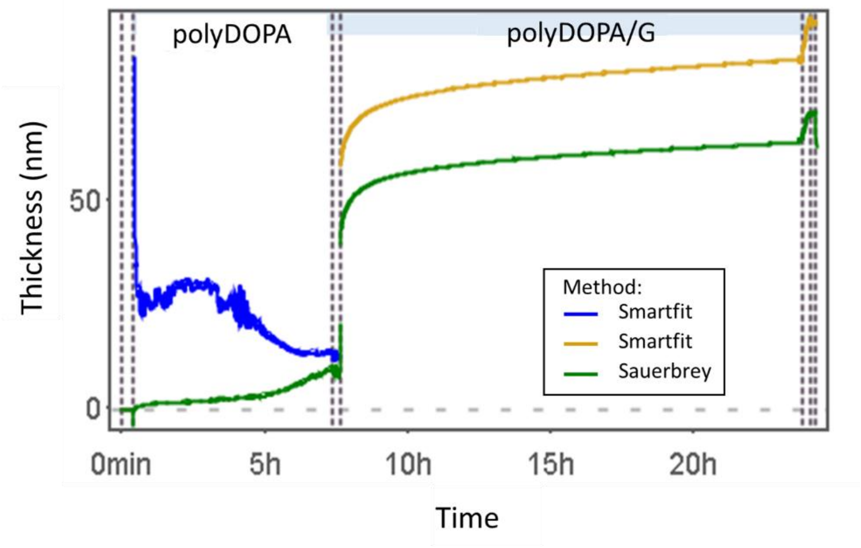


**Figure S5.** Comparison between layer thickness estimation using Sauerbrey model (green) and "Smartfit" method (blue and yellow).

*a-HCFs long-term culture*

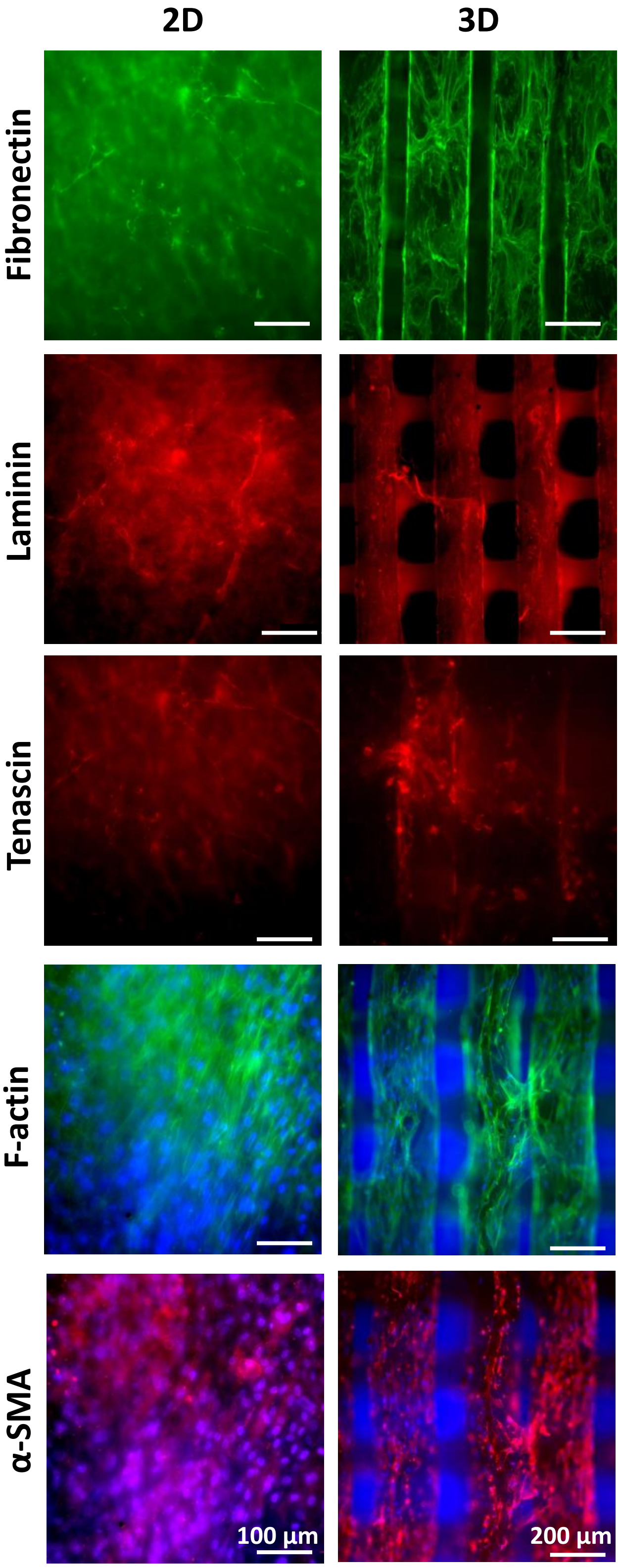

**Figure S6.** Immunostaining for F-actin, α-SMA, Fibronectin, Laminin, and Tenascin, performed after decellularization, on 2D and 3D PCL/polyDOPA/G scaffolds cultured for 3 weeks with a-HCFs isolated from atrial samples of patients with ischemic cardiomyopathy for *in vitro* modelling of human atrial fibrosis. Blue staining refers to nuclei in F-actin and α-SMA pictures.

### *In vitro cultures of v-HCFs on 2D and 3D scaffolds*

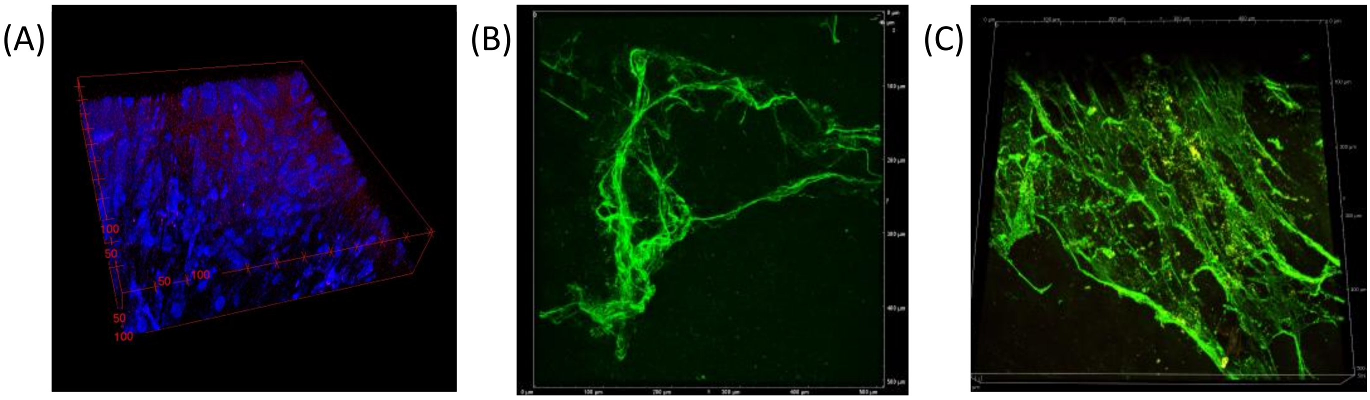


**Figure S7.** Two-photon microscopy images (TPEF) of 2D scaffolds: **(A)** 2D cellularized scaffolds reconstruction. Blue refers to DAPI staining of cell nuclei, red indicates α-SMA – Alexa555 immunostaining. **(B-C)** z-Stack measurement on the 2D decellularized scaffold stained for collagen I and collagen IV **(B)** and fibronectin and laminin **(C)**. Excitation wavelength was 950 nm. Signal acquired in the red channel (641 +/- 37.5 nm) integrates the tail of the two-photon excited fluorescence response of Alexa-555. Z-depth goes from 40 to 90 μm approximately.

**Movie S1**

**File: “alpha-SMA_in_3D_scaffold” = α-SMA in 3D scaffolds**

**Movie S1.** The presence of elongated shaped fibroblasts is clearly evident in this movie that reconstruct the 3D volume in real-time. The z-Stack measurement was recorded on 3D scaffolds labelled for α-SMA. Excitation wavelength was 950 nm. The blue channel (450 +/- 35 nm) clearly shows a weak fluorescence signal coming from the scaffold and from the cellular nuclei stained with DAPI. Signal acquired in the green channel (550 +/- 44 nm) integrates the two-photon excited fluorescence response of Alexa-555. Signal coming from the

stained protein within the cells is clearly observed, even from the very bottom (high value of Z-depth, ~250 μm) of the structure.

**Movie S2**

**File: "collagen_in_3D_scaffold" = collagen in 3D scaffolds**

**Movie S2.** The z-Stack measurement was recorded on 3D scaffolds labelled for collagen I and IV. Excitation wavelength was 950 nm. The blue channel (450 +/- 35 nm) clearly shows a weak fluorescence signal coming from the scaffold and from the residual fluorescence signal from the cellular nuclei (stained with DAPI). Signal acquired in the green channel (550 +/- 44 nm) integrates the two-photon excited fluorescence response of both labelling chromophores, precluding distinction of the two proteins (collagen I and IV). However, it is possible to clearly observe collagen I and IV signal coming from the whole scaffold, even from the very bottom (high value of Z-depth, ~300 μm) of the structure. Furthermore, below the top PCL filament we can observe residual structures of fibroblasts that have not been completely removed from the scaffold.

Videos link:
https://drive.google.com/drive/folders/14FJCprOUNhfQ8pnX_YmE8WBQPImANYn_?usp=share_link
